# Gut microbes mediate the synergistic effects of dietary cholesterol and saturated fat in driving fibrosing MASH

**DOI:** 10.1080/19490976.2026.2668121

**Published:** 2026-05-10

**Authors:** Jake B. Hermanson, Samar A. Tolba, Md Amran Gazi, Evan A. Chrisler, Manpreet Kaur, Ashley M. Sidebottom, Yongjun Liu, Guillermo Martinez-Boggio, Lauren N. Lucas, Daniel Amador-Noguez, Federico E. Rey, Vanessa A. Leone

**Affiliations:** a Department of Nutritional Sciences, University of Wisconsin-Madison, Madison, WI, USA; b Department of Nutrition and Clinical Nutrition, Faculty of Veterinary Medicine, Zagazig University, Zagazig, Egypt; c Department of Animal & Dairy Sciences, University of Wisconsin-Madison, Madison, WI, USA; d Duchossois Family Institute, The University of Chicago, Host-Microbe Metabolomics Facility, Chicago, IL, USA; e Department of Laboratory Medicine & Pathology, University of Washington, Seattle, WA, USA; f Department of Animal Science, University of California-Davis, Davis, CA, USA; g Department of Bacteriology, University of Wisconsin-Madison, Madison, WI, USA; h DOE Great Lakes Bioenergy Research Center, University of Wisconsin-Madison, Madison, WI, USA; i Department of Medical Microbiology and Immunology, University of Wisconsin-Madison, Madison, WI, USA

**Keywords:** MASLD, MASH, gut microbiome, diet, cholesterol, saturated fat, bile acid, fibrosis

## Abstract

Metabolic dysfunction-associated steatotic liver disease (MASLD) affects approximately one-third of the global population and can progress to metabolic dysfunction-associated steatohepatitis (MASH) with fibrosis, increasing the risk of cirrhosis, hepatocellular carcinoma, and mortality. Gut microbes driven by diets high in saturated fat, simple sugar, and cholesterol contribute to disease progression, yet the underlying mechanisms remain undefined. We explored the independent and synergistic effects of dietary saturated fat and cholesterol on MASH development using specific pathogen-free (SPF) and germ-free (GF) mice. We demonstrate that (1) both dietary cholesterol and saturated fat are required to induce fibrosing MASH in SPF mice, whereas GF mice are protected, (2) saturated fat and cholesterol individually alter gut microbial membership, potentially via altered bile acid metabolism, while their combination promotes a distinct composition, including an increase in *Parasutterella* spp. which correlates with hepatic fibrosis, and (3) diluted cecal contents from SPF, but not GF, mice fed high-fat, high-cholesterol diets are enriched in deoxycholic acid and activate human hepatic stellate cells *in vitro*, suggesting a mechanistic link between dietary lipid-induced microbiota and liver fibrogenesis. These findings reveal how specific Western dietary components shape the gut microbiota and contribute to hepatic fibrosis via stellate cell activation, offering potential targets for therapeutic interventions against MASLD/MASH.

## Graphical abstract

**Figure:**
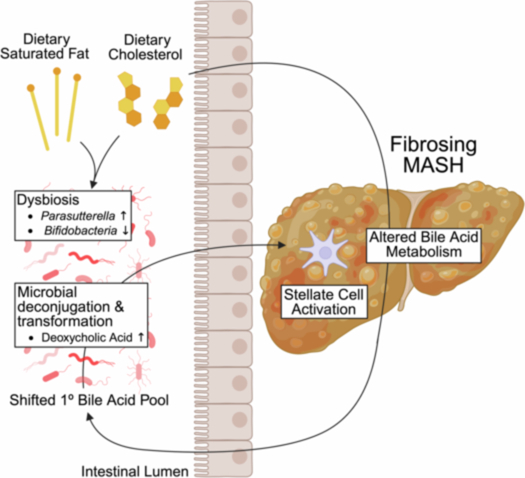

## Introduction

Metabolic dysfunction-associated steatotic liver disease (MASLD, formerly nonalcoholic fatty liver disease [NAFLD]^1^) is the most common chronic liver disease, impacting nearly a third of the population worldwide.^2^ Approximately 15%–20% of MASLD patients progress to metabolic dysfunction-associated steatohepatitis (MASH), which can lead to cirrhosis and hepatocellular carcinoma (HCC).^3^^,^^4^ MASH will soon become the leading indication for liver transplantation.^5^ Hepatic fibrosis, a hallmark of advanced MASH, is associated with worse disease outcomes. Higher fibrosis stages are associated with ~10 times greater risk of liver-related mortality.^6^ Despite a growing disease burden, treatment options remain limited. Resmetirom (marketed as Rezdiffra™) and semaglutide (marketed as Wegovy™), which were recently approved by the U.S. Food and Drug Administration as therapies for fibrosing MASH, achieved primary endpoints for fibrosis stage improvement in only a subset of patients, and their long-term efficacies are not fully understood.^7^^,^^8^ These limitations underscore the need for additional therapeutic interventions.

MASLD and MASH are heterogeneous diseases shaped by complex and dynamic interactions among genetic and environmental factors, including diet and the trillions of gut microbes that reside within the intestine.^9^ Deciphering the mechanistic connections between these factors is essential to advance our understanding of MASLD and MASH disease etiology for the development of effective, targeted interventions. Dietary cholesterol has emerged as a potent disease driver of MASLD and MASH pathogenesis. In both humans^10^^-^^16^ and preclinical animal models,^17^^-^^19^ elevated dietary cholesterol is strongly associated with disease prevalence and severity. For instance, in a large cohort of ~215,000 individuals, consuming ≥121.4 mg cholesterol per day increased MASLD risk, particularly among those with cirrhosis.^11^ In mice, dietary cholesterol is required to induce fibrosing MASH. While a high-fat diet (HFD) alone induces simple steatosis,^18^^,^^19^ incorporating 0.2% cholesterol (wt/wt) drives inflammation, fibrosis, and progression to HCC.^20^ It has been posited that cholesterol exacerbates steatosis and lipotoxicity, which in turn promote hepatic inflammation and eventually fibrosis.^21^ Despite this, the role of cholesterol in disrupting the gut‒liver axis, particularly through its interaction with gut microbes, remains poorly understood. Disentangling the specific contributions of dietary cholesterol and saturated fat is challenging in humans because of their co-occurrence in animal products such as red meat and eggs.

Gut microbes are known to influence metabolic diseases, including MASLD/MASH. Germ-free (GF) mice are generally protected from diet-induced obesity and liver pathology,^22^^,^^23^ whereas microbial transplantation from affected specific pathogen-free (SPF) mice can transfer pathological features to GF recipients.^20^^,^^24^ For example, long-term feeding of high saturated fat and cholesterol diet in mice leads to sequential development of steatosis, fibrosis, and HCC relative to chow or HFD feeding.^20^ This dietary intervention was associated with marked alterations in serum metabolites, including increased taurocholic acid (TCA) and decreased 3-indolepropionic acid (IPA). These changes in metabolites were accompanied by shifts in gut microbes, including enrichment of *Mucispirillum* and *Desulfovibrio* and depletion of *Bifidobacteria*. *In vitro* studies have shown that TCA promotes hepatic lipid accumulation, while IPA leads to reductions, suggesting that gut microbe-derived metabolites mediate key pathways in MASLD/MASH progression.^20^ While these findings implicate microbial metabolites in steatosis, their contribution to fibrogenesis, as well as the distinct roles of dietary cholesterol vs. saturated fat, remain unclear.

Gut microbes can enzymatically transform dietary cholesterol into non-absorbable coprostanol and other metabolites,^25^^-^^28^ thereby altering the homeostasis of host cholesterol metabolism. Studies by Le et al. and Yao et al. revealed that certain bacteria, e.g., *Bifidobacterium pseudolongum*, *Enterococcus*, and *Parabacteroides*, can contribute to either direct metabolism of exogenous cholesterol via various enzymes, e.g., sulfotransferases, or perform uptake and assimilation.^27^^,^^28^ These studies and others underscore the complex interplay between dietary lipids and gut microbes in liver disease pathogenesis. In this study, we sought to determine how dietary cholesterol vs. saturated fat independently and synergistically reshaped the gut microbiota composition in the context of MASLD/MASH progression using a murine model. We hypothesized that while each of these components alters the gut microbiota and host physiology in unique ways, their combination is required to induce fibrosing MASH in SPF mice, whereas their GF counterparts would remain largely protected against disease. Consistent with this hypothesis, we show that the combination of dietary cholesterol and saturated fat is essential to drive gut microbiota imbalances and disrupted bile acid metabolism, which together promote hepatic stellate cell (HSC) activation and progression to fibrosing MASH.

## Materials and methods

### Animals

All animal procedures were approved by the UW-Madison Institutional Animal Care and Use Committee (IACUC), protocol A006367. Eight-week-old male SPF C57Bl/6J mice (*n* = 6/group) were purchased from the Jackson Laboratories (barrier facility MP15) and maintained on a 12:12 hour light:dark cycle. From 8 to 16 weeks of age, the mice were housed 4/cage and provided autoclaved aspen shavings (Waldschmidt & Sons, Madison, WI), water, and LabDiet® 5k67 chow. Bedding was mixed twice weekly across all the cages to normalize the microbiome composition as previously described.^29^ Male GF C57Bl/6 mice (*n* = 4–7/group; some mice lost due to mortality) were bred in flexible film gnotobiotic isolators (CBC Clean, Inc., Madison, WI) at the University of Wisconsin–Madison Gnotobiotic Core facility and provided the same autoclaved aspen shavings, water, and LabDiet® 5K67 chow from 8 to 16 weeks of age. GF status was confirmed via 16S rRNA PCR on freshly collected fecal pellets weekly and via routine fecal cultivation under anaerobic and aerobic conditions. At 16 weeks of age, both SPF and GF mice were housed 2/cage and randomly assigned to one of six diets: low-fat (LF), low-fat + high-cholesterol (LFHC), low-fat + very high-cholesterol (LFVHC), high-fat (HF), high-fat + high-cholesterol (HFHC), or high-fat + very high-cholesterol (HFVHC) (diet composition and saturated vs. unsaturated fat content shown in Table S1). We initiated the dietary intervention at 16 weeks of age, as early middle-aged mice exhibit reduced metabolic flexibility and increased susceptibility to metabolic dysfunction and MASLD/MASH.^30^^,^^31^ This timing also enabled us to complete 8 weeks of mixed bedding prior to intervention, ensuring comparable baseline microbiota across cages.^29^ All the mice were maintained on these diets and provided with 18.1 g/L glucose + 23.1 g/L fructose in their drinking water for the entirety of the study period. Fresh feces and plasma (via the submandibular vein) were collected at baseline and every 4 weeks from SPF mice and every 8 weeks from GF mice and stored at −80 °C. Body weight, as well as food and water consumption, were measured weekly. After 8 and 24 weeks on diet, the mice were euthanized via CO_2_ asphyxiation. Portal and cardiac blood samples were collected into heparinized tubes, and 5 × 5 × 5 mm liver sections were prepared for histology. The remaining liver tissue was flash-frozen and stored at −80 °C. Epididymal, mesenteric, retroperitoneal, inguinal adipose tissue, and cecal luminal contents were weighed, flash-frozen, and stored at −80 °C.

### ALT measurement

~Two hundred microliters of blood was collected via the submandibular vein into heparin-coated microfuge tubes, followed by centrifugation (10,000 × *g*) for 10 minutes at 4 °C to obtain plasma. The plasma was diluted ¼ in phosphate-buffered saline (PBS), and alanine transaminase (ALT) activity was measured colorimetrically on a Catachem Well-T AutoAnalyzer using the ALT Dual Kit (Catachem, Oxford, CT).

### Total bile acid quantification

Fecal pellets were weighed, dried overnight at 55 °C in glass tubes, and reweighed to obtain dry weights. A total of 2 mL Folch solution (2:1 chloroform:methanol) was added, and the samples were incubated in a 60 °C water bath while shaking (~40 rpm) for 30 minutes. The samples were centrifuged at 1500 × *g* for 10 minutes. The lower chloroform phase was transferred to a new 1.5 mL centrifuge tube and evaporated under a N_2_ gas stream. The resulting lipids were resuspended in 100 µL of 1% Triton X-100 in EtOH. Total bile acid concentration in the fecal lipid extracts and cell-free cecal homogenates was determined using the Crystal Chem Mouse Total Bile Acids Assay Kit (Crystal Chem, Elk Grove Village, IL) according to the manufacturer's instructions.

### Liver histology

For Oil Red O staining, liver sections were cryopreserved in O.C.T. (Tissue-Tek), and 10 µm sections were cut using a Leica cryotome. Sections were fixed with 4% paraformaldehyde (PFA) for 15 minutes and stained with Oil Red O (lipid droplets) and hematoxylin (nuclei).^32^ Liver histology of formalin-fixed tissue was performed at the UW-Madison Translational Research Initiatives in Pathology (TRIP) laboratory. Briefly, following fixation in 4% neutral buffered formalin for 24 hours, the tissue was transferred to 70% EtOH, paraffin-embedded, and 5 µm sections were cut via a microtome. Following deparaffinization, the sections were stained with H&E, Picrosirius Red, or Masson's Trichrome. The MASLD activity score (MAS) was determined on H&E sections by a trained pathologist, blinded to subject treatment, according to Kleiner et al.^33^ based on hepatic steatosis, lobular inflammation, and hepatocyte ballooning. Lipid droplets (Oil-Red-O) and collagen deposition (Picrosirius Red, Masson's Trichrome) were quantified using ImageJ 2 (v 1.53a). Briefly, for Oil Red O- and Picrosirius red-stained sections, raw images were split into red, green, and blue grayscale channels. The green channel was selected, and a color threshold was set to highlight the stained areas. For Masson's Trichrome, raw images were split into a “Lab” grayscale stack, the “b” channel was selected, and a color threshold was set to highlight stained areas.

### RNA extraction and quantitative real-time reverse transcription PCR (qRT-PCR)

RNA was extracted from ~15 mg of liver tissue using TRIzol reagent and chloroform as previously described.^33^ cDNA was prepared using the iScript™ gDNA Clear cDNA Synthesis Kit (Bio-Rad, Hercules, CA) following the manufacturer's protocol. cDNA was combined with SYBR Green qPCR Master Mix (Bio-Rad, Hercules, CA) and forward and reverse primers (Table S2), and quantification of each gene was obtained on a CFX384 Real-Time PCR Detection System (Bio-Rad, Hercules, CA). The data were normalized using *glyceraldehyde-3-phosphate dehydrogenase* (*Gapdh*) as the housekeeping gene and presented as 2^(−ΔΔCt)^, with week 8 SPF LF set as the control.

### 16S rRNA gene amplicon sequencing and analysis

DNA was extracted from feces and cecal contents as previously described.^29^ The V4 region of the 16S rRNA gene was amplified using 515F-806R primers (Table S1). PCR amplification was performed at 94 °C for 3 minutes, followed by 40 cycles at 94 °C (45 seconds), 50 °C (60 seconds), and 72 °C (90 seconds). Paired-end reads (150 × 150 bp) of the resulting amplicons were sequenced on an Illumina MiSeq at Argonne National Laboratory. A total of 13,949,738 (fecal samples) and 2,804,624 (cecal samples) raw reads were obtained, with an average value of 35,952 (fecal samples) or 36,902 (cecal samples) reads per sample. Paired-end demultiplexed reads were imported and filtered utilizing Quantitative Insights Into Microbial Ecology (QIIME2, 2024.2)^34^ and trimmed to 120 bp. The divisive amplicon denoising algorithm (DADA2)^35^ was used to filter and denoise the imported demultiplexed sequences (via q2-dada2), where a total of 12,619,444 (fecal samples) and 2,453,434 (cecal samples) reads passed quality checks, with an average of 32,524 (fecal samples) and 32,282 (cecal samples) reads/sample. All samples were rarefied to a sequencing depth of 15,000 sequences per sample. α- and β-diversity metrics and Principal Coordinate Analysis (PCoA) were performed using the q2-diversity plugin in R. Taxonomy was assigned to amplicon sequence variants (ASVs) using the Silva-138 99% reference sequences via the q2-feature-classifier.

### Cell culture

LX-2 human immortalized hepatic stellate cells (Sigma-Aldrich, St. Louis, MO) were grown in Dulbecco's Modified Eagle Medium (DMEM, Thermo Fisher Scientific, Waltham, MA) supplemented with 2% fetal bovine serum (FBS, Thermo Fisher Scientific, Waltham, MA), 1000 U/mL penicillin, 1000 µg/mL streptomycin (Pen/Strep, Thermo Fisher Scientific, Waltham, MA), and 20 mM glutamine (Thermo Fisher Scientific, Waltham, MA) in a cell culture incubator set to 37ºC, 5% CO_2_, 90-95% relative humidity.^36^^,^^37^ Prior to experiments, cells were seeded in 12-well (~1 × 10^5^ cells/well) or 6-well (~3 × 10^5^ cells/well) tissue culture-treated cell culture plates (Corning, Corning, NY) and incubated for 24 hours or until ~80% confluent. Serum starvation was then performed to normalize the cell cycle across all wells by replacing the media with 0.2% FBS media for 24 hours.

Cell-free cecal homogenates were prepared by suspending the cecal contents in sterile PBS at 50 mg/mL. The samples were homogenized using a pellet pestle cordless motor (Thermo Fisher Scientific, Waltham, MA) for three rounds of 30 seconds. The homogenates were centrifuged at 10,000 × *g* (4 °C), and the supernatant was passed through a 0.22 µm filter and stored at −80 °C. Once the LX-2 cells reached ~80% confluence, the wells were washed twice with sterile PBS, and pre-warmed media containing cell-free cecal homogenate (10% vol/vol) were added. In separate experiments, deoxycholic acid (Thermo Fisher Scientific, Waltham, MA) was added to the growth media at the indicated concentrations. After four hours, the media was removed, the cells were washed twice with sterile PBS, and RNA was extracted using a RNeasy Mini Kit (Qiagen, Germantown, MD) according to the manufacturer's instructions. cDNA preparation and qRT-PCR were performed as described above.

### Fecal bile acid measurements

Lipidomics was performed at the Duchossois Family Institute at the University of Chicago. Metabolites were extracted from a subset of fecal samples from each group by adding 1 mL of 80% methanol (spiked with internal standards) per 100 mg of feces and homogenized at 4 °C on a Bead Mill 24 Homogenizer (1.6 m/s, six 30-second cycles, 5 seconds off between samples). The homogenates were centrifuged at 20,000 × *g* (−10 °C, 15 minutes), and the supernatant was collected. A total of 75  µL of the supernatant was added to autosampler vials, dried via a nitrogen stream (30 L/min on top; 1 L/min on bottom, 30 °C), and resuspended with a thermomixer (4 °C, 1000 rpm, 15 minutes) in 750 µL of 50% methanol. Insoluble debris was removed via centrifugation (4 °C, 20000 × g, 15 minutes), and the supernatant was transferred to an autosampler vial.

Metabolites were analyzed using a liquid chromatograph (Agilent 1290 Infinity II) coupled to a quadrupole time-of-flight (QTOF) mass spectrometer (Agilent 6546) in negative mode with an Agilent Jet Stream electrospray ionization source. A total of 5 µL of sample was injected into an XBridge© BEH C18 column (3.5 µm, 2.1 × 100 mm; Waters Corporation) with an XBridge© BEH C18 guard (Waters Corporation) at 45 °C. Elution began with 72% A (water, 0.1% formic acid) and 28% B (acetone, 0.1% formic acid) at 0.4 mL/minute for 1 minute and increased to 33% B over a 5-minute period, and then increased to 65% B over a 14-minute period. The flow rate was increased to 0.6 mL/minute and increased to 98% B over a 30-second period. This condition was held constant for 3.5 minutes. The flow rate was decreased to 0.4 mL/minute, and 28% B was added for 3 minutes to re-equilibrate. Electrospray ionization conditions were set (capillary voltage: 3.5 kV, nozzle voltage: 2 kV, detection window: 100–1700 *m/z*) with continuous infusion of reference masses (Agilent ESI TOF Biopolymer Analysis Reference Mix). A calibration curve (10-point) was used for quantitation, and the data were analyzed using MassHunter Profinder Analysis (v B.10, Agilent Technologies) and validated by comparison to authentic standards. For bile acids that were outside the range of the standard curve, normalized peak areas were calculated by dividing the raw peak areas of the target analytes by the average raw peak areas of the internal standards. Z-scores were determined for each sample within a given bile acid by the following formula:

$$Ζ=\frac{\chi -\mu }{\sigma },$$

where χ is the normalized peak area of the sample, μ is the mean peak area for a given bile acid across all samples, and σ is the standard deviation for a given bile acid across all samples.

### Cecal homogenate bile acid measurements

Cecal homogenates were analyzed using an ultrahigh-pressure liquid chromatography‒tandem mass spectrometry (uHPLC‒MS/MS) system consisting of a ThermoScientific Vanquish uHPLC system coupled with a heated electrospray ionization (HESI; using negative polarity) and hybrid quadrupole high-resolution mass spectrometer (Q Exactive Orbitrap; Thermo Scientific). The settings for the ion source were: auxiliary gas flow rate of 10, sheath gas flow rate of 30, sweep gas flow rate of 1, 2.5 kV spray voltage, 320 °C capillary temperature, 300 °C heater temperature, and S-lens RF level of 50. Nitrogen was used as a nebulizing gas by the ion trap source. Liquid chromatography (LC) separation was achieved using a Waters Acquity UPLC BEH C18 column with a 1.7 μm particle size and a length of 2.1 × 100 mm. Solvent A was water with 10 mM ammonium acetate adjusted to pH 6.0 with acetic acid. Solvent B was 100% methanol. The total run time was 31.5 min with the following gradient: a 0–24 min gradient from 30% solvent B (initial condition) to 100% solvent B; held 5 min at 100% solvent B; dropped to 30% solvent B for 2.5 min re-equilibration to initial conditions. The flow rate was 200 μL/min throughout. Other LC parameters were as follows: autosampler temperature, 4 °C; injection volume, 10 μL; and column temperature, 50 °C. The MS method performed a full MS1 full-scan (290–1000 m/z) together with a series of PRM (parallel reaction monitoring) scans. Untargeted experimental MS data were converted to mzXML format and used for targeted bile acid identification using El-MAVEN and matching sample peaks to standard peaks.^38^ Bile acids were quantified using 8-point external standard curves, with each bile acid ranging from 0.03125 to 4 μM, allowing for conversion of the raw signal to μM concentration. Samples were run at both 1/10 and 1/100 dilutions for all bile acid measurements to fall within the external standard signal range. The detection limit was less than 0.01 μM for all bile acids. The threshold for reported core bile acid transformation was 0.01 μM. Standards were purchased from Avanti Polar Lipids and dissolved and stored in methanol at −80 °C. See Table S3 for bile acid standard names and structural features.

### Statistical analyses

Statistical analyses were performed using GraphPad Prism (v 10.2.3) or R (v 4.4.2). Unless otherwise stated, the data are presented as the means ± standard error of the mean (SEM). See Table S4 for descriptions of the statistical tests used throughout the manuscript. The group sizes were *n* = 6 for all SPF groups and *n* = 4–7 for all GF groups. Outliers were identified using Grubbs' outlier test (*α* = 0.05) and removed prior to downstream analyses. Details regarding outliers are reported in Table S4. Only statistically significant *p*-values are shown in the figures.

For multi-way ANOVAs, if a significant higher-order interaction was detected between factors, *p*-values for the associated main effects and lower-order interactions are not reported in the figures, as these effects cannot be interpreted independently of the significant higher-order interaction. For significant effects, effect sizes (*R*^2^ or partial *η*^2^ [*η*^2^*p*]) and 95% confidence intervals are reported in Table S5.

Permutational multivariate analysis of variance (PERMANOVA) using ADONIS was performed on distance-based β-diversity matrices (Bray–Curtis, weighted UniFrac, unweighted UniFrac) to assess the main effects and interactions of time, cholesterol, and fat. Microbiome multivariable associations with linear models 2 (MaAsLin2)^39^ were performed using relative abundance feature tables obtained from QIIME2 to identify differential ASVs between treatment groups. The reference level was set to “LF,” and only significant (*p* < 0.05) associations are shown.

Linear regression modeling of fecal amplicon sequences vs. fibrosis was performed using the nlme package (v 3.1-165) in R:

$$\mathrm{Fibrosis}={\mathrm{ASV}}_{i}+\mathrm{Mouse}+e,$$

where Fibrosis is the percent area stained with Picrosirius Red at 24 weeks, ASV is the relative abundance of (1−i) ASVs, Mouse is the random effect of each animal, and e is the random residual effects. An autoregressive variance‒covariance matrix was applied to account for repeated measures within the mice. Only ASVs that showed a significant effect (*p* < 0.05) on fibrosis at each time point are shown.

## Results

### SPF and GF mice show similar energy balance and body composition across diets over time

**Figure 1.**
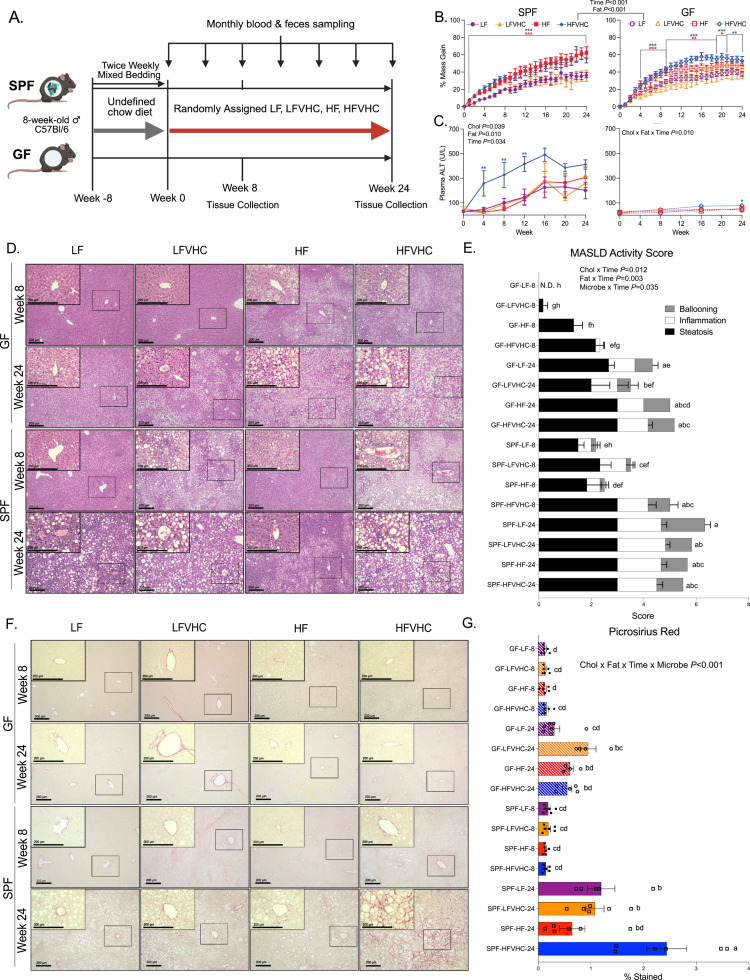
HFVHC diet drives fibrosing MASH in SPF but not GF mice. (A) Experimental schematic created on Biorender.com. (B) Percent body mass gain over time relative to baseline in SPF (left) and GF (right) mice. The data were analyzed via four-way repeated measures (RM) ANOVA (factors: cholesterol, fat, microbes, and time), followed by Tukey's multiple comparisons within timepoint. (C) Plasma alanine transaminase (ALT) levels were analyzed via three-way RM ANOVA (factors: cholesterol, fat, and time) within the SPF and GF groups, followed by Tukey's multiple comparisons within timepoint. For panels B and C, *p < 0.05, **p < 0.01, and ***p < 0.005. The asterisk color represents which group is significantly different from the corresponding LF control. (D) Representative H&E-stained liver sections; scale bar = 200 µm. The inset images (400×) correspond to the boxed regions in 100× images. (E) Total MASLD activity score (MAS) split by components. (F) Representative picrosirius red-stained liver sections; scale bar = 200 µm. The inset images (400×) correspond to the boxed regions in 100× images. (G) Quantification of percent area stained (indicating collagen). The data were analyzed via four-way ANOVA (factors: cholesterol, fat, microbes, and time), followed by Tukey's multiple comparisons. Bars with the same letter are not significantly different (*p* > 0.05). *n* = 4–7/group (see Table S4 for outliers). Low-fat (LF); low-fat + very high-cholesterol (LFVHC); high-fat (HF); high-fat + very high-cholesterol (HFVHC).

We first investigated how gut microbes interact with two prominent Western dietary components, cholesterol and saturated fat, in shaping MASLD/MASH outcomes. To test this, 16-week-old male GF and SPF C57Bl/6 mice were provided fructose- and glucose-supplemented drinking water and fed *ad libitum* one of four semi-purified diets: low-fat (LF), low-fat +  very high-cholesterol (LFVHC), high-fat (HF), or high-fat +  very high-cholesterol (HFVHC) (Table S1) for 8 or 24 weeks (Figure 1A).

Both SPF and GF mice fed HF or HFVHC exhibited increased percent body mass gain relative to baseline, demonstrating a significant main effect of dietary fat level and time (fat *p* < 0.001, time *p* < 0.001, respectively, Figure 1B). Percent body mass gain was evident throughout the study in both SPF HF- and HFVHC-fed mice, whereas their GF counterparts showed a delay in weight gain, achieving significance after 4 weeks (Figure 1B). However, there was no overall effect on microbial status, suggesting that SPF and GF mice gain body mass similarly in response to dietary fat and cholesterol (Figure 1B). Total caloric intake mirrored these findings. In SPF mice, total caloric intake was influenced by an interaction between fat and time (fat × time *p* < 0.001) as well as between cholesterol and fat (cholesterol × fat *p* = 0.043) (Figure S1A). In GF mice, a significant main effect of cholesterol (cholesterol *p* = 0.028) and a fat × time interaction (fat × time, *p* = 0.030) was detected (Figure S1A). This finding indicated that dietary cholesterol alone contributed to increased caloric intake independent of saturated fat or time on the diet. Similar to that observed for body mass gain (Figure 1B), microbial status did not affect caloric intake throughout the study (Figure S1A). Together, these data suggest that SPF and GF mice exhibit similar body weight gain and food consumption, regardless of dietary saturated fat or cholesterol level.

We next examined indicators of body composition, including liver and white adipose tissue mass. Liver mass, expressed as a percentage of final body mass (LM%BM), was significantly influenced by interactions between dietary cholesterol and fat (cholesterol × fat *p* = 0.001) and between fat, microbes, and time (fat × microbe × time *p* < 0.001) (Figure S1B). These results indicate that diet-induced increases in LM%BM, a gross indicator of MASLD, occur in both GF and SPF mice and become more pronounced over time.

Expansion of peripheral adipose tissue, another hallmark of MASLD, was assessed by summing the masses of four major WAT depots (inguinal, retroperitoneal, mesenteric, and epididymal) and expressing the total as a percentage of final body mass (WAT%BM). Significant interactions were observed between dietary cholesterol and microbe status (cholesterol × microbe *p* < 0.001), fat and microbe status (fat × microbe *p* < 0.001), and fat and time (fat × time *p* = 0.001) (Figure S1C). Importantly, HFVHC-fed SPF and GF mice exhibited similar WAT%BM at both 8 and 24 weeks, suggesting that GF mice gained comparable levels of adiposity to SPF mice (Figure S1C), which is consistent with similar overall body mass gain between SPF and GF groups throughout the study (Figure 1B).

Taken together, these data demonstrate that SPF and GF mice exhibited comparable caloric intake, body mass gain, and expansion of both liver and WAT in response to dietary fat and cholesterol intake. While some temporal differences were observed, particularly a delay in weight gain in GF mice, the overall patterns converged over time, indicating that gross metabolic responses to diet were independent of gut microbes.

### The presence of high-fat- and cholesterol-induced gut microbes is required for diet-induced fibrosing MASH

We next examined parameters of liver function and histological indicators of disease in SPF and GF mice. SPF mice fed HFVHC exhibited significantly elevated circulating alanine transaminase (ALT) levels as early as 4 weeks relative to all other groups, which persisted through week 12 (cholesterol *p* = 0.039, fat *p* = 0.010, time *p* = 0.034) (Figure 1C, left panel). Conversely, ALT elevation in HFVHC-fed GF mice was modest and delayed, reaching significance after 24 weeks compared to LF-fed GF counterparts (cholesterol × fat × time *p* = 0.010) (Figure 1C, right panel).

Histology was assessed in H&E-stained liver sections via MASLD activity score (MAS), which integrates scores of steatosis, lobular inflammation, and hepatocyte ballooning as previously described.^40^ Representative images are shown in Figure 1D. Time was a major driver of MAS, showing significant interactions with dietary cholesterol (cholesterol × time *p* = 0.012; *η*^2^*p* = 0.08 [0.01, 1.00]), saturated fat (Fat × time *p* = 0.003), and microbial status (Microbe ​​​​​​× time *p* = 0.035) (Figure 1E). After 8 weeks, GF mice displayed elevated steatosis in HF and HFVHC groups but showed minimal evidence of inflammation or ballooning (Figure 1E). In contrast, SPF mice exhibited both steatosis and inflammation, particularly in response to LFVHC and HFVHC diets (Figure 1E). Importantly, HFVHC-fed SPF mice had significantly higher MAS at 8 weeks compared with HF- or LF-fed SPF mice, as well as all GF groups at the same time point (Figure 1E). By the end of the study, MAS had increased across all groups in both SPF and GF conditions (Figure 1E).

Oil Red O (ORO) staining of the liver sections revealed significant interactions between saturated fat and time (fat × time *p* < 0.001) and between cholesterol, fat, and microbial status (cholesterol × fat × microbe *p* = 0.007) (Figure S1D, E). After 8 weeks, steatosis in GF mice was strongly influenced by saturated fat, with HF- and HFVHC-fed GF mice showing significantly higher ORO staining than their LF- and LFVHC-fed counterparts (Figure S1E). In SPF mice, the inclusion of high dietary cholesterol tended to increase hepatic steatosis after 8 weeks (Figure S1E), suggesting differential patterns of diet-induced lipid accumulation between SPF and GF mice. Consistent with MAS, all the groups showed elevated ORO staining at 24 weeks (Figure S1E). These data indicate that both SPF and GF mice are equally susceptible to diet-induced weight gain and hepatic lipid accumulation, with cholesterol intake contributing to early increases in MAS regardless of fat level. However, only HFVHC-fed SPF mice developed early and progressively more severe clinical markers of liver injury, as reflected by increased ALT levels over time, highlighting a microbe-dependent effect in driving disease progression to MASH.

We next assessed hepatic fibrosis, a hallmark of advanced MASH, under both GF and SPF conditions using histological analyses. Picrosirius red staining of liver sections showed a significant four-way interaction (cholesterol × fat × time × microbe *p* < 0.001). Pairwise comparisons showed that HFVHC-fed SPF mice at 24 weeks exhibited significantly increased hepatic fibrosis compared with all other groups (Figure 1F, G). Masson's Trichrome produced consistent results, with collagen deposition significantly higher in HFVHC-fed SPF mice at 24 weeks compared with all other groups (cholesterol × fat × time × microbe *p* < 0.001) (Figure S1F, G). Fibrosis staging was not included owing to limited reproducibility across blinded scoring assessments, which is consistent with known constraints of semiquantitative fibrosis evaluation.^41^^,^^42^

Taken together, these data indicate that dietary fat and cholesterol synergize to accelerate disease progression in a microbiota-dependent manner, with substantial hepatic fibrosis manifesting only in HFVHC-fed SPF mice after 24 weeks.

### Dietary cholesterol promotes proinflammatory and fibrosis-related gene expression signatures in SPF mice

**Figure 2.**
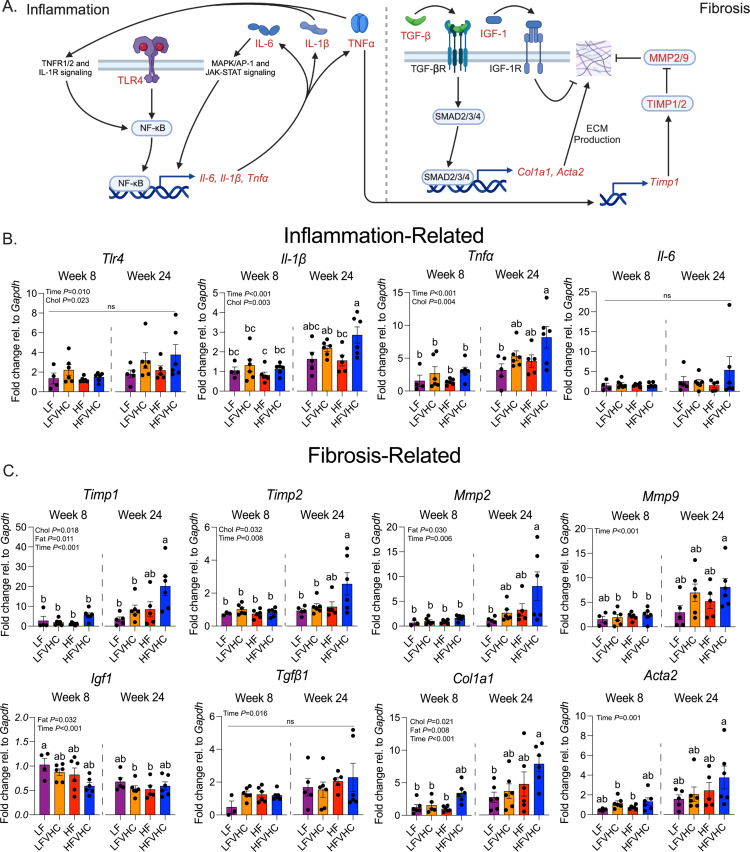
Dietary cholesterol enhances the expression of inflammation- and fibrosis-related genes in SPF mice. (A) Schematic of key signaling pathways involved in inflammation and fibrosis in MASH, created on Biorender.com. The expression of the genes analyzed in panels B and C is highlighted in red. (B and C) Expression levels of inflammation-related (B) and fibrosis-related (C) genes normalized to those of *glyceraldehyde 3-phosphate dehydrogenase* (*Gapdh*) and shown as fold-change relative to LF-fed SPF mice at 8 weeks, as determined via the 2^−^^∆∆Ct^ method. The data represent means ± SEM and were analyzed via three-way ANOVA (factors: cholesterol, fat, and time), followed by Tukey's multiple comparisons. Bars with the same letter are not significantly different (*p* > 0.05). *n* = 3–6/group (see Table S4 for outliers). Low-fat (LF); low-fat + very high-cholesterol (LFVHC); high-fat (HF); high-fat + very high-cholesterol (HFVHC); tumor necrosis factor superfamily member 1A/2 (TNFR1/2); interleukin-1 receptor (IL-1R); Toll-like receptor 4 (TLR4); nuclear factor-kappa B (NF-κB); mitogen-activated protein kinase (MAPK); activator protein-1 (AP-1); Janus kinase (JAK); signal transducer and activator of transcription (STAT); tumor necrosis factor-alpha (*Tnfα*/TNFα); interleukin 1-beta (*Il-1β*/IL-1β); interleukin-6 (*Il-6*/IL-6); transforming growth factor-beta (receptor) (TGF-*β*[R]); suppressor of mothers against decapentaplegic 2/3/4 (SMAD2/3/4); insulin-like growth factor-1 (receptor) (IGF-1[R]); *Collagen type I alpha I chain* (*Col1a1*); *Alpha-actin 2* (*Acta2*); extracellular matrix (ECM); matrix metalloproteinase 2/9 (MMP2/9); tissue inhibitor of metalloproteinase 1/2 (TIMP1/2).

To explore the molecular response to high dietary cholesterol and saturated fat in SPF mice, we examined hepatic expression levels of key pro-inflammatory and fibrogenic genes. As outlined in Figure 2A (left panel), *Toll-like receptor 4* (*Tlr4*), a pattern recognition receptor that is implicated in MASLD/MASH,^43^ as well as downstream pro-inflammatory cytokines, including *Tumor necrosis factor-alpha* (*Tnfα*), *Interleukin-1 beta* (*Il-1β*), and *Interleukin-6* (*Il-6*), are known to be upregulated in MASH patients.^44^ Hepatic expression of *Tlr4*, *Il-1β*, and *Tnfα* increased ~2-3-fold in HFVHC-fed SPF mice relative to LF-fed controls after 24 weeks, with significant main effects of both time and cholesterol (Time *p* = 0.010, <0.001, and <0.001; cholesterol *p* = 0.023, 0.003, and 0.004, respectively). In contrast, *Il-6* expression was not significantly altered under these conditions (Figure 2B).

Given that proinflammatory cytokines drive HSC activation and fibrogenesis,^45^ we next measured fibrosis-related gene expression. For example, as shown in Figure 2A (right panel), TNFα and IL-1β have been shown to increase the expression of *tissue inhibitor of metalloproteinase 1* (*Timp1*),^46^ which, along with matrix metalloproteinases (MMPs), contributes to the regulation of extracellular matrix (ECM) remodeling in the liver. We observed main effects of cholesterol, fat, and time (*p* = 0.018, 0.011, and <0.001, respectively) on *Timp1* expression, which was significantly increased in HFVHC-fed SPF mice after 24 weeks compared to all other groups (Figure 2C). A similar pattern was observed for *Timp2*, with main effects of cholesterol (*p* = 0.032) and time (*p* = 0.008). HFVHC-fed SPF mice showed significantly higher *Timp2* expression than all other groups except the HF-fed SPF mice. The main effects of fat (*p* = 0.030) and time (*p* = 0.006) were evident for *Mmp2*, with pairwise comparisons showing that HFVHC-fed SPF mice at 24 weeks had higher expression than LF-fed mice after 24 weeks and all groups at week 8 (Figure 2C). In contrast, *Mmp9* expression was influenced only by time (*p* < 0.001), with HFVHC-fed mice showing significantly higher levels at 24 weeks than LFVHC-, HF-, and HFVHC-fed mice at 8 weeks (Figure 2C).

Additionally, pro-fibrotic pathways in the liver are partly driven by transforming growth factor beta (TGF-β), the protein product of *Tgfβ1*, which promotes the expression of *collagen type I alpha I chain* (*Col1a1*) and *alpha-actin 2, smooth muscle* (*Acta2*). Conversely, insulin-like growth factor-1 (IGF-1) is proposed to protect against hepatic fibrosis through several mechanisms (Figure 2A). We observed a time-dependent increase in *Tgfβ1* expression (*p* = 0.016), though no significant pairwise differences were apparent (Figure 2C). The expression of *Igf1* was significantly impacted by the main effects of saturated fat (*p* = 0.032) and time (Time *p* < 0.001), whereas LFVHC- and HF-fed mice at week 24 showed reduced *Igf1* expression relative to LF-fed mice at week 8 (Figure 2C). *Col1a1* expression was increased by dietary cholesterol, saturated fat, and time (*p* = 0.021, 0.008, <0.001, respectively). HFVHC-fed mice at week 24 showed significantly higher *Col1a1* expression relative to LF-fed mice at week 24, as well as LF-, LFVHC-, and HF-fed mice at week 8 (Figure 2C). *Acta2* expression was impacted only by time (*p* = 0.001), with HFVHC-fed mice at week 24 exhibiting higher expression than LFVHC- and HF-fed mice at week 8 (Figure 2C).

These data suggest that localized hepatic gene expression of growth factors in the liver involved in regulating fibrosis is influenced more by time and saturated fat than by dietary cholesterol. Specifically, *Tgfβ1* increases while *Igf1* decreases over time, with saturated fat contributing to an overall decrease in hepatic *Igf1* expression. However, it is important to consider that key regulators such as TGF-β are also produced by nonparenchymal cells, such as immune cells and endothelial cells, which were not directly evaluated in this study.^47^ These data, coupled with histological outcomes, indicate that dietary cholesterol-driven inflammation emerges early in SPF mice, while fibrosis develops over time and is further modulated by dietary saturated fat, which appears to be necessary, but not sufficient, on its own to drive fibrosis (Figures 1F, G; and S1F, G).

### Gut microbiota diversity and composition are altered by dietary cholesterol and saturated fat during the development and progression of MASH

The composition of the gut microbiota is rapidly altered in response to dietary modifications,^48^ and our findings indicate their crucial role in triggering diet-induced fibrosing MASH. To examine the impact of dietary cholesterol vs. saturated fat on gut microbiota composition, we performed 16S rRNA gene amplicon sequencing. To ascertain the dose-dependent effect of dietary cholesterol, we included two additional diets: low-fat high-cholesterol (LFHC) and high-fat high-cholesterol (HFHC), each of which contain 0.2% cholesterol (Table S1).

We first assessed overall microbial community membership via α-diversity (within-sample diversity) metrics in feces collected every 4 weeks. Reductions in richness (Chao1) and richness/evenness (Fisher's alpha) were evident as early as 4 weeks on HFVHC diet (cholesterol × fat × time *p* < 0.001 for both; Figure S2A). Both the Shannon index, another richness/evenness metric, and the Simpson index, a measure of species dominance, were altered by week 8 (time *p* < 0.001, cholesterol × fat *p* < 0.001 for both; Figure S2A).

Examination of α-diversity in cecal contents collected after 8 and 24 weeks showed that Chao1 richness was reduced in response to diet, driven by an interaction between cholesterol and saturated fat (cholesterol × fat *p* = 0.048), demonstrating a consistent trend toward reduced richness in HFVHC-fed mice (Figure S2B). A similar trend was observed for Fisher's alpha, though statistical significance was not achieved (Figure S2B). The Shannon index was affected by both time and a cholesterol × fat interaction (time *p* < 0.001, cholesterol × fat *p* = 0.031), while the Simpson index increased, indicating lower diversity, due to the main effects of saturated fat and cholesterol (fat *p* = 0.001, cholesterol *p* = 0.010; Figure S2B). The latter findings contrast with those observed in feces (Figure S2A), suggesting regional differences in HFVHC-induced changes in α-diversity.

We next investigated β-diversity metrics between samples to determine how diet altered microbial community membership across time. In feces, nonphylogenetic-based Bray‒Curtis analysis showed significant interactions between cholesterol and saturated fat (cholesterol × fat *p* = 0.001), cholesterol and time (cholesterol × time *p* = 0.003), and saturated fat and time (fat × time *p* = 0.002), indicating that these dietary components dynamically shift the fecal microbiota composition over time (Figure S2C). These interactions were evident at week 4 (*p* = 0.001) and persisted at all subsequent time points, including week 8 (*p* = 0.004), 12 (*p* = 0.001), 16 (*p* = 0.003), 20 (*p* = 0.045), and 24 (*p* = 0.019) (Figure S2D). Importantly, HFVHC-fed mice demonstrated a pronounced and persistent separation from all other groups beginning at week 8, revealing that the combination of saturated fat and cholesterol drove a distinct microbial composition throughout the study (Figure S2D).

**Figure 3.**
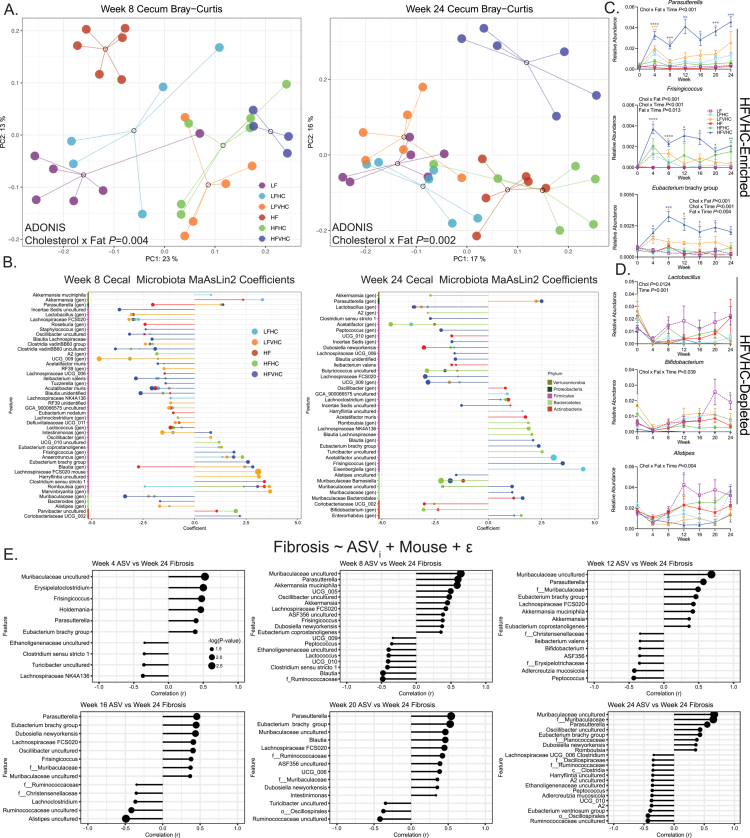
Dietary cholesterol and saturated fat lead to rapid and sustained shifts in gut microbiota that are associated with hepatic fibrosis. (A) Bray–Curtis β-diversity PCoA of the cecal microbiota after 8 (left panel) and 24 (right panel) weeks. The dots represent individual mice, and the open circles represent centroids with lines connecting individual dots within a treatment group. Data were analyzed via two-way ADONIS (factors: cholesterol, fat). (B) Amplicon sequence variants (ASVs) were significantly enriched or depleted relative to LF-fed mice via multivariate MaAsLin2 analysis. Positive coefficients indicate enrichment; negative coefficients indicate depletion. (C and D) Relative abundance of fecal ASVs enriched (C) or depleted (D) in response to HFVHC over time. The data are presented as the means ± SEMs and were analyzed via three-way ANOVA (factors: cholesterol, fat, and time) followed by Tukey's multiple comparisons within timepoint. *p < 0.05, **p < 0.01, ***p < 0.005, and ****p < 0.001. The asterisk color represents significance relative to LF-fed mice. (E) Linear modeling of the relationships between fecal ASVs in feces collected every 4 weeks throughout the study relative to the severity of hepatic fibrosis (based on the Picrosirius red-stained area) determined at 24 weeks. *r*, Pearson correlation coefficient; the dot size corresponds to −log(*p*-value). For B and E, the dot size reflects the −log(*p*-value). *n* = 4–6/group (samples removed due to rarefaction; see Materials and Methods and Table S4). “(gen)” refers to genus-level annotation. Low-fat (LF); low-fat + high-cholesterol (LFHC); low-fat + very high-cholesterol (LFVHC); high-fat (HF); high-fat + high-cholesterol (HFHC); high-fat + very high-cholesterol (HFVHC); amplicon sequence variant (ASV).

To determine the regional influence of saturated fat vs. cholesterol over time, we examined β-diversity of cecal microbes at 8 and 24 weeks (Figures 3A; S2E, F). At both time points, ADONIS analysis showed microbiota community membership was significantly altered by interactions between saturated fat and cholesterol, as indicated by shifts in both non-phylogenetic-based (Bray–Curtis: 8 weeks *p* = 0.004; 24 weeks *p* = 0.002; Figure 3A) and phylogenetic-based (unweighted: 8 weeks *p* = 0.018; 24 weeks *p* = 0.001; Weighted: 8 weeks *p* = 0.007; 24 weeks *p* = 0.021; Figure S2E, F) distance metrics.

To identify specific ASVs that contribute to the observed shifts in microbial composition, we performed Microbiome Multivariable Associations with Linear Models 2 (MaAsLin2).^39^ After 8 weeks, several ASVs were enriched in HFVHC-fed mice, including *Parasutterella*, *Intestinimonas*, *Frisingicoccus*, *Anaerotruncus*, *Eubacterium* brachy group, *Blautia*, *Romboutsia*, and *Coriobacteriaceae* UCG_002 (Figure 3B, left panel). Conversely, LF-fed mice showed enrichment of taxa such as Ruminococcaceae uncultured, *Lactobacillus*, *Lachnospiraceae* FCS020, *Staphylococcus*, *Oscillobacter* uncultured, *Blautia* uncultured, *Clostridia* vandin BB60 uncultured, *Acetatifactor muris*, *Ileibacterium valens*, *Tuzzerella*, *Acutalibacter muris*, *Blautia* unidentified, *Muribaculaceae*, and *Bacteroides* (Figure 3B, left panel).

Interestingly, several ASVs enriched by HFVHC diet, including *Parasutterella*, *Frisingicoccus*, *Eubacterium* brachy group, and *Blautia*, remained elevated after 24 weeks, while *Lachnoclostridium*, Muribaculaceae, and *Enterorhabdus* emerged only at this later timepoint (Figure 3B, right panel). LF-fed mice continued to show enrichment of Ruminococcaceae uncultured, *Lactobacillus*, and *Lachnospiraceae* FCS020, whereas additional taxa, including *Clostridium* sensu stricto 1, *Peptococcus*, Ruminococcaceae, *Lachnospiraceae* UCG_006, *Blautia* unidentified, *Ileibacterium valens*, *Butyricicoccus* uncultured, UCG_009, *Alistipes* uncultured, and *Barnesiella*, were exclusively enriched at 24 weeks (Figure 3B, right panel).

Pairwise comparisons of cecal ASVs between diets at 8 and 24 weeks via MaAsLin2 revealed differential and time-dependent effects of cholesterol vs. saturated fat on microbial community membership. The addition of very high cholesterol to the HF diet dramatically impacted ASV relative abundances at both timepoints (Figure S3A, B), which is consistent with our findings related to β-diversity (Figures 3A; S2C–E). Importantly, *Parasutterella* was the only ASV consistently enriched in response to high dietary cholesterol, regardless of the saturated fat level or timepoint (Figure S3A-D). Further, *Bifidobacterium* was reduced in HFVHC-fed mice relative to HF only after 24 weeks (Figure S3B), indicating a delayed response to dietary cholesterol (Figure S3A). Conversely, *Lactobacillus* was reduced in LFVHC-fed mice relative to LF at both timepoints; however, no differences were observed between HF- and HFVHC-fed mice. This may suggest that high dietary saturated fat intake masks the influence of cholesterol on *Lactobacillus* abundance over time (Figure S3A, B). When examining the effects of saturated fat alone, i.e., in the absence of added cholesterol, more pronounced shifts in the cecal microbial composition were evident at 8 weeks than at 24 weeks (Figure S3C, D). Although few taxa showed consistent patterns at both timepoints, *Ileibacterium valens* was reduced at 8 and 24 weeks, whereas *Bifidobacterium* and *Lactobacillus* were both reduced in response to saturated fat after 24 weeks (Figure S3D).

To better understand how these microbial shifts identified via MaAsLin2 evolved throughout the course of disease, we examined the relative abundances of key microbial taxa in fecal samples collected monthly (Figure 3C). This analysis revealed that many diet-induced shifts were observed as early as 4 weeks. The HFVHC diet enriched *Parasutterella*, *Frisingicoccus*, and *Eubacterium* brachy group, which persisted throughout the study (Figure 3C). Other taxa, such as *Lactobacillus*, *Bifidobacterium*, and *Alistipes,* were robustly depleted in HFVHC-fed mice relative to LF-fed controls (Figure 3D).

Finally, linear modeling was applied to fecal ASVs across time at 4, 8, 12, 16, 20, and 24 weeks to identify taxa that significantly correlated with fibrosis severity based on Picrosirius red staining at 24 weeks (Figure 1G). Notably, several taxa identified by MaAsLin2 as enriched in HFVHC-fed mice, including *Parasutterella*, *Frisingicoccus*, *Eubacterium* brachy group, and members of *Muribaculaceae*, also showed an early and sustained association with hepatic fibrosis, suggesting that these microbes may act as disease drivers (Figure 3E).

These microbial shifts raise important questions regarding how dietary interventions influence host-microbiota metabolic interactions, particularly bile acid (BA) metabolism, which plays a central role in gut‒liver axis regulation. Notably, *Parasutterella*, which was enriched early in HFVHC-fed mice and associated with more severe fibrosis, belongs to the Proteobacteria phylum, members of which are generally more tolerant of high BA concentrations.^49^ The introduction of *Parasutterella* mc1 to a complex gut microbial community has been shown to decrease the cecal levels of cholic acid (CA), taurocholic acid (TCA), taurodeoxycholic acid (TDCA), 7-ketoDCA, and glycolithocholic acid (GLCA) sulfate in the cecum, suggesting that this genus may directly participate in BA metabolism and/or alter the abundance/activity of other BA-metabolizing microbiota.^50^ In contrast, *Bifidobacteria* and *Lactobacillus*, which were depleted under these dietary conditions, are more sensitive to elevated BA concentrations,^49^ and possess well-established capabilities to perform deconjugation and transformation of BAs within the gut.^51^^,^^52^ Together, these observations highlight the complex interplay between microbial composition, BA dynamics, and liver health. Accordingly, we next investigated how dietary cholesterol and saturated fat shape BA metabolism over time.

### High dietary cholesterol and saturated fat reshape host bile acid metabolism, signaling, and fecal bile acid profiles over time in SPF mice

**Figure 4.**
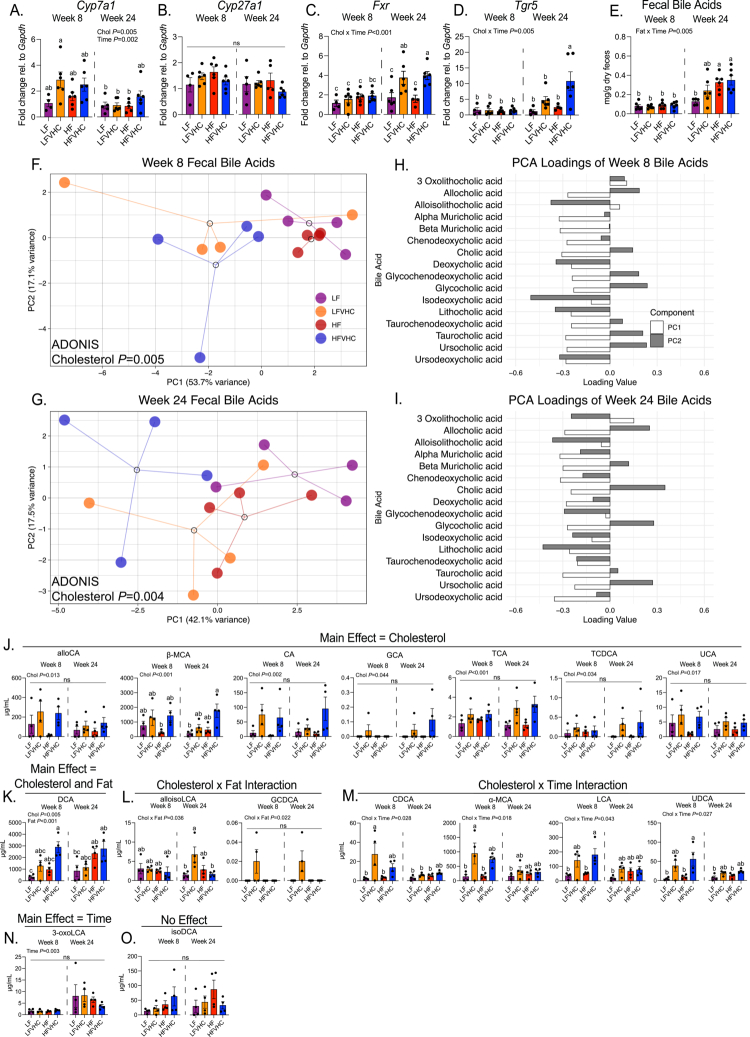
Time- and diet-dependent remodeling of fecal bile acid (BA) composition by cholesterol and saturated fat in SPF mice. (A–D) Hepatic expression of genes involved in bile acid (BA) biosynthesis and sensing shown as fold-change relative to LF-fed SPF mice at 8 weeks, normalized to *glyceraldehyde 3-phosphate dehydrogenase* (*Gapdh*) and determined via the 2^–∆∆Ct^ method. *Cytochrome P450 family 7 subfamily A member 1* (*Cyp7a1*) (A); *Cytochrome P450 family 27 subfamily A member 1* (*Cyp27a1*) (B); *Farnesoid x receptor* (*Fxr*) (C); and *Takeda G protein-coupled receptor 5* (*Tgr5*) (D). (E) Fecal BA concentrations in SPF mice. The data represent the means ± SEMs and were analyzed via three-way ANOVA (factors: cholesterol, fat, and time), followed by Tukey's multiple comparisons. Bars with the same letter are not significantly different (*p* > 0.05). (F and G) Principal component analysis (PCA) plots of fecal BA from SPF mice at 8 weeks (F) and 24 weeks (G). The dots represent individual mice, and the open circles represent centroids with lines connecting individual dots, which were analyzed via two-way ADONIS (factors: cholesterol and fat). (H, I) PC loadings of individual BA after 8 weeks (H) and 24 weeks (I). (J–O) Quantification of BA in the feces of SPF mice at 8 and 24 weeks significantly impacted by a main effect of dietary cholesterol (J), the main effects of dietary cholesterol and saturated fat (K), the interaction between dietary cholesterol and saturated fat (L), the interaction between dietary cholesterol and time (M), the main effect of time (N), or not impacted by any factor (O). Data represent the means ± SEMs and were analyzed via three-way ANOVA (factors: cholesterol, fat, and time), followed by Tukey's multiple comparisons. Bars with the same letter are not significantly different (*p* > 0.05). *n* = 4–6/group for panels A–E (see Table S4 for outliers). *n* = 4/group for panels F–O (subset of mice selected for BA analyses). Low-fat (LF); low-fat + very high-cholesterol (LFVHC); high-fat (HF); high-fat + very high-cholesterol (HFVHC); cholic acid (CA); Muricholic acid (MCA); glycocholic acid (GCA); taurocholic acid (TCA); taurochenodeoxycholic acid (TDCA); ursocholic acid (UCA); deoxycholic acid (DCA); lithocholic acid (LCA); glycochenodeoxycholic acid (GCDCA); chenodeoxycholic acid (CDCA); ursodeoxycholic acid (UDCA).

BAs are synthesized in the liver from cholesterol and are key components in lipid digestion and absorption. In addition, they play a central role in maintaining the gut‒liver axis through their signaling functions and interactions with gut microbes. Given the significant shifts observed in gut microbes in SPF mice, we examined how dietary cholesterol and saturated fat affect the host hepatic expression of genes involved in BA metabolism. We first assessed the hepatic expression of genes that encode key enzymes involved in host BA synthesis, mainly *cytochrome P450 family 7 subfamily A member 1* (*Cyp7a1*), which catalyzes the rate-limiting step in the classic BA biosynthetic pathway, as well as *cytochrome P450 family 27 subfamily A member 1* (*Cyp27a1*), which is associated with the alternative BA biosynthetic pathway. We noted that dietary cholesterol and time significantly impacted *Cyp7a1* expression (cholesterol *p* = 0.005; time *p* = 0.002) (Figure 4A). LFVHC- and HFVHC-fed mice exhibited numerically elevated expression of *Cyp7a1* at 8 weeks, although these increases did not reach statistical significance at this timepoint (Figure 4A). Interestingly, *Cyp7a1* in LFVHC-fed mice at 8 weeks was significantly increased relative to LF, LFVHC, and HF-fed groups at week 24; however, no differences were observed among groups within the 24-week timepoint (Figure 4A), suggesting a transient impact of dietary cholesterol on BA synthesis genes. No significant differences were observed in *Cyp27a1* gene expression across all groups or timepoints, indicating a limited role for the alternative pathway in this context (Figure 4B). This finding suggests that high dietary cholesterol intake exerts early effects on host BA metabolism mediated by the classic biosynthesis pathway, which is lost following long-term exposure.

Given that BA synthesis is tightly regulated by feedback signaling through BA-responsive receptors, we next assessed hepatic expression of the nuclear BA receptor *Farnesoid X receptor* (*Fxr*) and the membrane G-protein-coupled BA receptor *Takeda G protein-coupled receptor 5* (*Tgr5*).^53^ *Fxr* expression was significantly influenced by an interaction between time and dietary cholesterol (time × cholesterol *p* < 0.001) (Figure 4C). After 24 weeks, high dietary cholesterol significantly increased *Fxr* expression, with both HFVHC- and LFVHC-fed mice exhibiting higher expression relative to all other groups, except for HFVHC-fed mice at 8 weeks (Figure 4C).

Similarly, *Tgr5* expression was significantly affected by the interaction between time and dietary cholesterol (time × cholesterol *p* = 0.005) (Figure 4D). Pairwise comparisons revealed significantly increased *Tgr5* expression exclusively in HFVHC-fed SPF mice at 24 weeks relative to all other groups (Figure 4D).

Together, these findings suggest that while high dietary cholesterol transiently increases *Cyp7a1* expression early during exposure, prolonged cholesterol intake is associated with increased expression of BA-sensing receptors involved in feedback inhibition, metabolic adaptation, and inflammatory modulation.^53^ The induction of *Fxr* and *Tgr5* at later timepoints is consistent with a shift from BA production toward enhanced BA signaling and homeostatic regulation, potentially reflecting host adaptation to sustained elevations in luminal and microbially modified BA pools.

We next examined total fecal BA concentrations and individual BA species. An interaction between fat and time (fat × time *p* = 0.005) elicited a significant impact on total fecal BA levels, where HF- and HFVHC-fed mice at 24 weeks exhibited higher concentrations compared to all other groups, except LFVHC at 24 weeks, a group that showed intermediate levels (Figure 4E). These data suggest that while cholesterol influences BA synthesis, saturated fat levels may alter BA excretion and composition over time.

Lipidomic analysis of fecal BAs was performed in a subset of SPF mice at 8 and 24 weeks, showing that of the 40 identified BAs, 16 were quantifiable with distinct patterns influenced by dietary cholesterol and fat. A heatmap of all 40 detected BAs (including both primary and secondary as well as conjugated and unconjugated species) is shown in Figure S4A. Principal component analysis (PCA) of the 16 quantified BAs revealed that dietary cholesterol significantly altered BA profiles at both 8 (*p* = 0.005) and 24 weeks (*p* = 0.004) (Figure 4F, G). PC loading plots were assessed to identify specific BAs driving these compositional shifts (Figure 4H, I). At 8 weeks, α-Muricholic acid (α-MCA), β-Muricholic acid (β-MCA), chenodeoxycholic acid (CDCA), deoxycholic acid (DCA), isoDCA, lithocholic acid (LCA), and ursodeoxycholic acid (UDCA) exhibited negative loadings on both PC1 and PC2 (Figure 4H), indicating stronger associations with HFVHC-fed mice, contributing to the separation of this group in the ordination plot (Figure 4F). At 24 weeks, allocholic acid (alloCA), β-MCA, CA, glycocholic acid (GCA), taurocholic acid (TCA), and ursocholic acid (UCA) showed similar loading outcomes (Figure 4H, I).

Absolute quantifications of these BAs in feces are shown in Figure 4J–O. While saturated fat alone did not elicit a significant main effect on any BA, a significant main effect of cholesterol was observed for several species, including alloCA (*p* = 0.013), β-MCA (*p* < 0.001), CA (*p* = 0.002), GCA (*p* = 0.004), TCA (*p* < 0.001), and taurochenodeoxycholic acid (TCDCA) (*p* = 0.034) (Figure 4J). The fecal β-MCA concentration was higher in HFVHC-fed mice compared to both LF-fed mice after 24 weeks and HF-fed mice after 8 weeks (Figure 4J). DCA levels were significantly impacted by both cholesterol (*p* = 0.005) and fat (*p* < 0.001), whereas HFVHC-fed mice exhibited the highest fecal DCA concentrations relative to LF-fed mice at both 8 and 24 weeks (Figure 4K). Further, significant interactions between cholesterol and fat were evident for both alloisoLCA (*p* = 0.036) and glycochenodeoxycholic acid (GCDCA) (*p* = 0.022) (Figure 4L). Pairwise comparisons showed that LFVHC-fed mice had significantly higher alloisoCA compared to both LF- and HFVHC-fed counterparts at 24 weeks (Figure 4L). Other BAs showed significant interactions between cholesterol and time. For instance, α-MCA was significantly impacted (*p* = 0.018), where LFVHC-fed mice at 8 weeks showed higher levels than LF- and HF-fed mice at 8 weeks, as well as LF-fed mice at 24 weeks (Figure 4M). Likewise, CDCA (*p* = 0.028) was increased in LFVHC-fed mice at 8 weeks compared to all groups except for HFVHC-fed mice at both 8 and 24 weeks (Figure 4M). Both LCA (*p* = 0.043) and UDCA (*p* = 0.027) showed similar trends, with the highest concentrations observed in mice fed HFVHC at 8 weeks (Figure 4J). 3-oxoLCA was increased over time, regardless of diet (time *p* = 0.003; Figure 4K), while isoDCA was not differentially impacted by factors of diet or time (Figure 4L).

Together, these data show that prolonged intake of high dietary cholesterol and saturated fat synergistically remodel the BA pool in SPF mice. Given the well-established link between BAs, gut microbes, and hepatic inflammation and fibrosis, these shifts may contribute to the fibrotic phenotype observed only in SPF mice after prolonged HFVHC feeding.

### Gut microbiota-dependent components modulated by HFVHC diet activate human hepatic stellate cells *in vitro*

**Figure 5.**
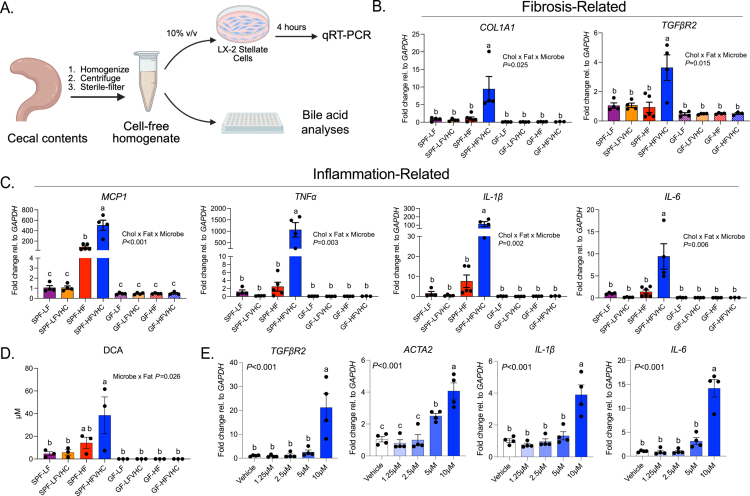
Microbiota-dependent gut factors from HFVHC-fed SPF mice induce pro-fibrotic and pro-inflammatory activation of hepatic stellate cells (HSC) *in vitro*. (A) Experimental schematic created on Biorender.com; cecal contents were pooled from 3 randomly selected mice/group after 24 weeks on the diet, homogenized, centrifuged, and sterile-filtered, followed by bile acid quantification. LX-2 HSC cells were exposed to homogenates at 10% media (v/v) for 4 hours, followed by qRT-PCR. (B and C) Gene expression of fibrosis-related (B) and inflammation-related (C) HSC activation markers. (D) Concentration of deoxycholic acid (DCA) in cecal homogenates. (E) LX-2 HSC cells were treated with the indicated concentrations of DCA or vehicle for 4 hours, followed by qRT-PCR. Alpha-smooth muscle actin (*ACTA2*). The data represent the means ± SEM. (B–D) Data were analyzed via three-way ANOVA (factors: time, fat, and microbe) followed by Tukey's multiple comparisons within a timepoint. (E) Data were analyzed via one-way ANOVA followed by Tukey's multiple comparisons. Bars with the same letter are not significantly different (*p* > 0.05). The gene expression values (C, E) were normalized to those of *GAPDH*, determined via the 2^–∆∆Ct^ method and expressed relative to those of the SPF-LF (B, C) or vehicle (E) groups. *n* = 3‒5/group, representative of 3 independent experiments. Low-fat (LF); low-fat + very high-cholesterol (LFVHC); high-fat (HF); high-fat + very high-cholesterol (HFVHC); *collagen type I alpha I chain* (*COL1A1*), *monocyte chemoattractant protein-1* (*MCP1*), *interleukin-6* (*IL-6*), *transforming growth factor-beta receptor type 2* (*TGFβR2*), *tumor necrosis factor-alpha* (*TNFα*), *interleukin 1-beta* (*IL-1β*).

Since we observed shifts in the gut microbiota and BAs in response to high levels of dietary cholesterol and saturated fat that coincided with the development of fibrosing MASH, we next tested whether gut-derived factors induced by HFVHC feeding could directly drive fibrogenic processes. To address this, cell-free cecal homogenates, i.e., cecal water, were generated from GF and SPF mice fed LF, LFVHC, HF, or HFVHC diet for 24 weeks. These homogenates were applied to human-derived LX-2 HSCs *in vitro*, as outlined in Figure 5A. After 4 hours of exposure, the expression of both pro-fibrogenic and pro-inflammatory genes was measured via qRT-PCR. We observed a significant three-way interaction between dietary cholesterol, saturated fat, and the microbial status of cecal homogenates on *COL1A1* (*p* = 0.025) and *TGFβR2* (*p* = 0.015) (Figure 5B). Cecal homogenates from HFVHC-fed SPF mice drove a significant upregulation of *COL1A1* and *TGFβR2* expression by ~10- and ~4-fold, respectively, relative to all other groups (Figure 5B).

We next investigated the expression of pro-inflammatory markers in LX-2 HSCs following exposure to cecal homogenates. Similar to what was observed for pro-fibrotic genes, a significant three-way interaction between dietary cholesterol, saturated fat, and microbial status was observed for *Monocyte chemoattractant protein-1* (*MCP1*) (*p* < 0.001), *TNFα* (*p* = 0.003), *IL-1β* (*p* = 0.002), and *IL-6* (*p* = 0.006) (Figure 5C). Pairwise comparisons showed that cecal homogenates from HFVHC-fed SPF mice significantly increased the expression of all four activation markers relative to all other groups. Here, *MCP1*, *TNFα*, and *IL-1β* were robustly upregulated (~500-, 1200-, and 100-fold, respectively) in LX-2 HSCs exposed to HFVHC cecal homogenate, while *IL-6* showed a 10-fold increase (Figure 5C). Interestingly, *MCP1* expression was also significantly increased following exposure to cecal homogenate from HF-fed SPF mice relative to all groups except SPF-HFVHC (Figure 5C). These data suggest that HFVHC feeding induces gut factors that elicit strong activation of pro-fibrogenic and inflammatory pathways in HSCs *in vitro*, providing a mechanistic link between diet, gut microbes, and hepatic injury.

To gain further insights into microbially derived factors in HFVHC cecal homogenates that may drive the upregulation of pro-fibrotic and pro-inflammatory genes in LX-2 HSCs, we examined total bile acid (TBA) concentrations.^54^^,^^55^ We observed a significant three-way interaction between dietary cholesterol, fat, and microbial status (*p* < 0.001, Figure S5A). Surprisingly, cecal homogenates from LFVHC-fed SPF mice had the highest TBA levels, followed by homogenate from LF-fed SPF mice, both of which were significantly greater than all other groups (Figure S5A). These results suggest that the TBA concentration alone in cecal homogenates is not the main driver of HSC activation observed *in vitro*, but specific BAs may be enriched in HFVHC-fed SPF mice.

Analysis of BA species in cecal homogenates revealed an interaction between microbial status and fat (*p* = 0.026) for DCA (Figure 5D), where HFVHC-fed SPF mice exhibited significantly increased levels relative to all groups except for HF, although a numerical trend was evident. Importantly, because these samples were pooled for the *in vitro* LX-2 exposure experiments, cells treated with cecal homogenates from HFVHC-fed SPF mice were exposed to approximately threefold higher DCA concentrations than cells treated with homogenates from HF-fed SPF mice. A similar trend was observed for ω/α-MCA, which also showed a significant interaction between microbial status and fat content (*p* = 0.019; Figure S5B). While both GCA and TCDCA were also impacted by a significant interaction between fat and microbial status (*p* < 0.001 and *p* = 0.002, respectively), cecal homogenate from SPF LF- and LFVHC-fed mice showed higher levels relative to all other groups (Figure S5C). CA was impacted by both interactions between microbial status and fat (*p* < 0.001) as well as between microbial status and cholesterol (*p* = 0.032) (Figure S5D). CDCA (*p* = 0.015) and β-MCA (*p* = 0.008) also showed a significant interaction between microbial status and cholesterol, with cecal homogenates from LFVHC- and HFVHC-fed SPF mice having the highest levels (Figure S5E). Both LCA and UDCA were impacted by a main effect of microbial status (*p* = 0.002 and *p* < 0.001, respectively), where SPF mice showed higher levels than GF mice regardless of diet (Figure S5F).

Because DCA showed the most distinct pattern of significance in cecal homogenate from HFVHC-fed SPF mice, we next tested whether this BA could activate HSCs *in vitro*. LX-2 cells were exposed to various concentrations for 4 hours. We observed significantly increased expression of both fibrosis-related genes (*TGFβR2* and *ACTA2*) and inflammation-related genes (*IL-1β* and *IL-6*) in response to 10  µM DCA relative to vehicle control (Figure 5E). *ACTA2* expression was also modestly increased by 5 µM DCA (Figure 5E).

Taken together, these data suggest that TBA levels alone did not account for the observed HSC activation induced by cecal homogenates from HFVHC-fed SPF mice. However, fat- and cholesterol-induced microbially derived BA species, such as DCA, may serve as key drivers of the initial pro-inflammatory and fibrogenic activation of HSCs *in vitro.* Further work is needed to determine whether DCA acts alone or in concert with additional BAs or other microbially derived factors to activate HSCs. In addition, whether the sustained expansion of dietary cholesterol and fat-induced microbial community members, such as *Parasutterella*, directly contributes to increased DCA over time requires further exploration in the context of fibrosis in MASH.

## Discussion

In humans, fibrosing MASH develops through a heterogeneous and dynamic process, where multiple factors, including diet and the gut microbiota, have been established as robust disease moderators.^9^ However, the specific interactions between common Western dietary components and gut microbiota imbalances in the context of MASLD to MASH progression are not fully understood.^56^^-^^60^ Dietary cholesterol and saturated fat are often consumed together in human diets, making it difficult to disentangle their individual effects on the gut microbiota and disease progression. By independently manipulating these two prominent Western dietary components, our study revealed their distinct and synergistic effects on the gut microbiota composition and development of fibrosing MASH. Using a multifactorial design that incorporated dietary composition, microbial status (GF vs. SPF), and time, we were able to dissect both the individual and combined contributions of these variables to disease etiology in a well-established mouse model. Our findings support the notion that cholesterol and saturated fat remodel the gut microbiota early in disease, leading to persistent alterations that contribute to fibrosing MASH over time.

We showed significant induction of hepatic fibrosis in SPF mice fed HFVHC diet compared to all other groups (Figures 1F, G; S1F, G), suggesting that dietary saturated fat, cholesterol, and the presence of gut microbes are necessary for the development of MASH with fibrosis *in vivo*. This finding was further supported via the robust activation of LX-2 HSCs *in vitro* by cecal homogenate from HFVHC-fed SPF mice (Figure 5). These findings suggest that gut-derived factors induced by the synergistic effects of cholesterol and fat may directly promote hepatic fibrogenesis through HSC activation, which could be mediated by several mechanisms.^20^^,^^61-64^ Recent studies have begun to identify specific diet- and microbiota-derived metabolites that modulate fibrogenic pathways in MASH. For instance, Provera et al. demonstrated that ketogenic versus Western diets differentially shape gut microbes and attenuate liver inflammation and fibrosis, in part through enhanced β-hydroxybutyrate signaling that suppresses pro-inflammatory and fibrogenic signaling pathways.^65^ Marchianò et al. reported that alloLCA, a microbiota-derived secondary BA, exerts direct anti-fibrotic effects by modulating immune signaling and restoring BA and microbial homeostasis in experimental MASH.^66^ Others have shown that diets high in cholesterol (2% wt/wt) and fat (40% kcal) can increase the relative abundance of *Blautia producta,* a gut bacteria that produces 2-oleoylglycerol and activates hepatic macrophages in mice.^67^ A separate study showed that 2% wt/wt inclusion of cholesterol shifted gut microbiota and BA profiles, including increased hepatic DCA and CDCA, which were sufficient to activate inflammatory gene expression in HepG2 cells.^63^ Additional studies are needed to identify the factor(s) responsible for HSC activation and to test their sufficiency both *in vitro* and *in vivo*. Further, given the complexity of the *in vivo* hepatic microenvironment, future validation in primary or human HSCs as well as *in vivo* models is warranted to confirm the pro-fibrotic role of DCA and other gut-derived factors. Given the strong link between BA dysregulation and fibrogenesis, several BA-based therapeutic strategies have been explored for MASH. FXR and TGR5 agonists can reduce hepatic inflammation and fibrosis by restoring bile acid homeostasis and modulating lipid and glucose metabolism.^68^ Although synthetic FXR agonists such as obeticholic acid improve the histological features of MASH, their efficacy is modest and limited by side effects such as pruritus and dyslipidemia, leading to a lack of FDA approval.^68^ Our studies may support ongoing work that is focused on developing safer BA-based therapies for MASLD/MASH.

Several gut microbial taxa exhibited early and sustained enrichment in response to HFVHC diet and were positively associated with hepatic fibrosis at multiple time points (Figure 3E). *Parasutterella* was particularly sensitive to cholesterol and showed a strong association with hepatic fibrosis (Figure 3C, E). Colonization of SPF mice with *Parasutterella* mc1 has been shown to alter gut BA profiles and reduce the gene expression of several ileal BA transporters and FXR signaling pathway components, suggesting a role in regulating enterohepatic BA circulation.^50^ Additionally, during antibiotic-induced dysbiosis, *Parasutterella excrementihominis* upregulates fatty acid biosynthesis pathways in the small intestine, highlighting its metabolic adaptability and potential to thrive under disrupted microbial conditions.^69^ Despite the prevalence of this genus in humans and mice,^70^^,^^71^ it remains relatively unexplored. Some studies have linked *Parasutterella* with health benefits, including reduced hypothalamic inflammation and improved LDL levels.^70^^,^^72^ In contrast, other studies have associated increased *Parasutterella* abundance with hepatic steatosis,^73^ type 2 diabetes,^74^ and obesity.^75^ However, to our knowledge, no studies have identified an enrichment of *Parasutterella* in human MASH cohorts compared to healthy controls. In our study, *Parasutterella* was consistently enriched in mice with more severe hepatic fibrosis and rapidly expanded upon exposure to HFVHC feeding, prior to detectable inflammation or fibrosis. It is likely that *Parasutterella* exhibits strain-level heterogeneity that may explain its differential association with metabolic diseases. While *Parasutterella* was strongly associated with fibrosis and rapidly expanded in HFVHC-fed mice, further studies are needed to determine causality in human MASH.

In addition to direct interactions with sterols in the gut, both dietary cholesterol and saturated fat significantly impact BA metabolism and secretion.^76^ BAs, in turn, can exert both beneficial and detrimental effects on the gut microbiota community and may represent a key mechanistic link between diet, shifts in the gut microbiota, and fibrosing MASH. We observed significant alterations in the abundance of several BAs, predominantly driven by dietary cholesterol, even in the absence of saturated fat (Figures 4, S4C, and S5). DCA is a strongly hydrophobic and cytotoxic secondary BA^77^ produced by microbial dehydroxylation of CA. DCA levels tended to be the highest in HFVHC-fed mice at both 8 and 24 weeks, while CA was highest in LFVHC-fed mice after 8 weeks and in HFVHC-fed mice at both timepoints (Figure 4J, K). These findings suggest that increased levels of DCA are not solely dependent on CA availability, but rather on shifts in microbial capacity to metabolize CA.

These changes in BAs likely contribute to the depletion of certain microbial taxa. In our model, HFVHC diet feeding depleted *Lactobacillus*, *Bifidobacteria*, *Alistipes*, and other taxa negatively associated with hepatic fibrosis (Figure 3B–E). This trend is consistent with previous evidence suggesting that several members of *Bifidobacteria* and *Lactobacillus* are beneficial to human health^78^ and protect against liver disease and damage.^79^^-^^87^ The depletion of these bacteria could be due to the observed shifts in BAs, particularly unconjugated BAs,^88^ that negatively impact several members of *Bifidobacteria* and *Lactobacillus*. In our study, the fecal DCA and CA concentrations tended to be the highest in HFVHC-fed SPF mice (Figure 4J, K), suggesting that they may be partially responsible for the loss of BA-sensitive taxa. For instance, the growth of several *Bifidobacteria* species, including *B. longum*, *B. pseudolongum*, *B. adolescentis,* and *B. pseudocatenulatum*, was inhibited by CDCA, DCA, and CA *in vitro.*^49^

While BAs can differentially influence certain microbes, elevated DCA may also act directly on the liver to promote fibrosis. In our study, DCA levels were elevated in both feces and cecal homogenates from HFVHC- and HF-fed SPF mice (Figures 4K, 5D). We demonstrated that DCA activated LX-2 HSCs *in vitro*, with the most robust activation observed at physiologically relevant concentrations (10 µM) (Figure 5E). These findings suggest that DCA present in the gut may contribute to HSC activation following BA resorption back into the circulation. Importantly, DCA is increased in patients with MASH^89^^-^^92^ and has been implicated in MASLD and MASH through mechanisms that include the induction of pyroptosis and inflammation in hepatocytes.^62^^,^^63^ Although our results support a role for DCA in the activation of HSCs, future studies are needed to test whether DCA alone is sufficient to promote hepatic fibrogenesis.

Beyond BA sensitivity, the microbial shifts in response to HFVHC feeding observed in our study may also be explained in part by their ability to either assimilate, metabolize, or tolerate excess luminal cholesterol.^27^^,^^28^ For example, *Eubacterium coprostanoligenes*, which encodes the intestinal sterol metabolism A (*ismA*) enzyme responsible for converting cholesterol to coprostanol in the gut^26^ exhibited cholesterol-dependent enrichment in mice fed LFVHC and HFVHC diets (Figure S3A). On the other hand, *B. pseudolongum* has been previously shown to assimilate dietary cholesterol to a high degree *in vivo.*^27^ The *Bifidobacteria* ASV observed in our study that was depleted in response to cholesterol and saturated fat (Figure 3D) mapped with ~100% identity to *B. pseudolongum* (data not shown). Similarly, both glycosylation and dehydrogenation of cholesterol have been reported in several *Oscillibacter* members, a genus that also largely decreased in abundance in response to dietary cholesterol in our study (Figure S3A). Additional mechanistic studies, including those using gnotobiotic mice, will be needed to determine how these interactions shape gut microbiota community membership and function and whether they contribute to the development of fibrosing MASH.

Our studies present some limitations that warrant consideration. First, all the mice received glucose- and fructose-supplemented drinking water, which limited our ability to disentangle the independent contributions of simple sugars from those of dietary fat, cholesterol, and gut microbes in the context of MASLD/MASH development and progression. Importantly, uniform sugar supplementation may also have masked potential sugar‒lipid‒microbiome interactions that could differentially influence microbial ecology and disease severity. Notably, Shen et al. reported increased colonic *Parasutterella* relative abundance in response to high-fructose corn syrup,^93^ suggesting that the inclusion of glucose- and fructose-supplemented drinking water may have contributed to the observed *Parasutterella* enrichment observed here. Future studies are needed to identify whether simple sugars synergize with dietary fat and cholesterol to drive the changes in gut microbiota and the fibrosing MASH phenotypes observed in this study. Second, only male mice were used, which limits the generalizability of our findings regarding host‒microbe‒diet interactions to female mice and hence to human patient populations. Importantly, MASLD exhibits sex-dimorphic patterns, with a lower risk in premenopausal women compared to men, but a similar prevalence post-menopause due to complex sex-specific factors.^94^ While our *in vitro* studies demonstrated that gut-derived factors from HFVHC-fed SPF mice are sufficient to activate HSCs, and we identified DCA as a potential mediator (Figures 5; S5), it is likely that other BAs and microbial metabolites present in cecal homogenates also contribute to HSC activation.

Although we provide an extensive characterization of diet-induced shifts in the gut microbiota, the mechanisms driving these microbial shifts remain speculative and require further investigation. While our data demonstrate strong associations between diet-induced alterations in the gut microbiota and the development of fibrosing MASH, causality cannot be inferred from the current experimental design. Fecal microbiota transplantation (FMT) from HFVHC-fed SPF mice into GF recipients would be an important next step in determining whether microbes are sufficient to transfer fibrotic disease phenotypes. In addition, the distinct metabolic and immune characteristics of GF mice complicate the interpretation of their relative resistance to fibrosing MASH.^95^ Complementary studies in antibiotic-treated SPF mice may help isolate microbiota-dependent effects while minimizing developmental differences inherent to the GF state. While we did not directly assess intestinal inflammation in the present study, it is a recognized feature of MASLD/MASH in both humans and animal models.^96^ High-fat, high-cholesterol diet-induced changes in BA profiles likely disrupt intestinal homeostasis and contribute to shifts in microbial community composition, which is consistent with prior reports.^97^^,^^98^ Examination of BA signaling, particularly FXR-dependent enterohepatic feedback, may provide a mechanistic link between intestinal inflammation and microbiota dynamics, including microbiota-driven BA transformations.^99^ Finally, the identification of *Parasutterella* spp. as a microbe associated with more severe disease warrants further investigation. Monoassociation or defined community studies in GF mice could help delineate the specific contribution of *Parasutterella* to hepatic metabolic dysfunction and fibrogenesis and assess its potential relevance as a therapeutic target.

In conclusion, our work highlights the synergistic effects of dietary cholesterol and saturated fat in shaping the gut microbiota composition and their subsequent influence on hepatic fibrosis via gut-derived factors. Using a multifactorial design, we identified key aspects of host metabolic health, gut microbiota community membership, and fecal BA profiles that are differentially influenced by dietary components and microbial presence across the early and late stages of MASLD/MASH. Our findings suggest that specific gut-derived factors, including DCA, other BAs, and microbial taxa, such as *Parasutterella*, may play a critical role in activating HSCs and promoting fibrogenesis in MASH. These insights provide a foundation for future studies aimed at elucidating microbial and metabolic pathways involved in hepatic fibrosis, with the goal of developing microbiota- and diet-based strategies to prevent or reverse the MASH disease course.

## Supplementary Material

Table_S4.xlsx Table_S4.xlsx

Table_S5.xlsx Table_S5.xlsx

Table_S2.xlsx Table_S2.xlsx

Table_S1.xlsx Table_S1.xlsx

Supplementary Material Supplementary_Figures.docx

Table_S3.xlsx Table_S3.xlsx

## Data Availability

The data that support the findings of this study are available at https://10.6084/m9.figshare.29598581. 16S rRNA gene amplicon sequencing data are deposited in the National Center for Biotechnology Information (NCBI) Sequence Read Archive (SRA) BioProject ID PRJNA1289062.
