# Supplementary material for: Gut microbes mediate the synergistic effects of dietary cholesterol and saturated fat in driving fibrosing MASH
Source: Gut Microbes. 2026 May 10;18(1):2668121. doi: 10.1080/19490976.2026.2668121 (PMC13166192; doi:10.1080/19490976.2026.2668121)
Supplement: Supplementary Material — Supplementary_Figures.docx [file KGMI_A_2668121_SM6315.docx]

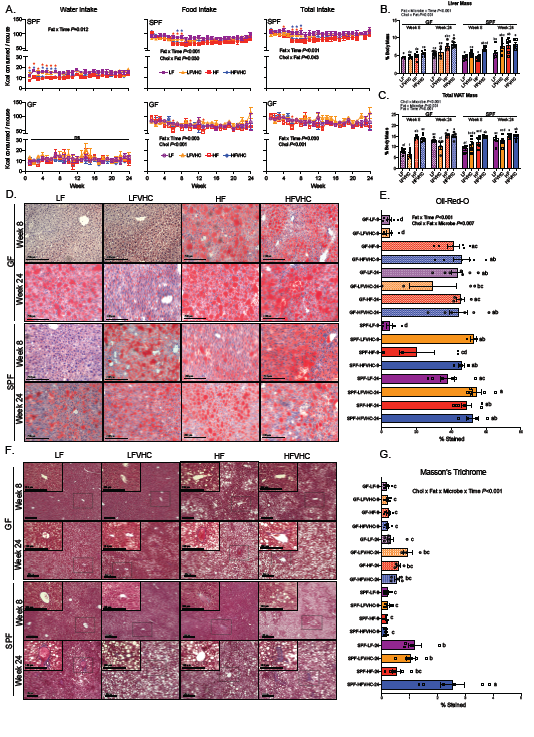


**Supplementary Figure 1. GF and SPF mice exhibit comparable caloric intake and adiposity, but disparate liver lipid and fibrosis profiles.**

**A)** Caloric intake attributed to water, food, or combined during the duration of the study. Data represent means ± SEM, analyzed via 4-way RM ANOVA (factors: Cholesterol, Fat, Microbes, Time) followed by Tukey’s multiple comparisons within timepoint. **P*<0.05. Asterisk color represents which group is significantly different from LF. **B,C)** Liver mass (B) and total white adipose tissue (WAT) mass (C) as a percent of body mass. Data represent means ± SEM analyzed via 4-way ANOVA (factors: Cholesterol, Fat, Microbes, Time), followed by Tukey’s multiple comparisons. Bars with the same letter are not significantly different (*P*>0.05). **D)** Representative Oil Red O-stained liver sections; scale bar=200µm. Inset images (400x) correspond to boxed area in 100x images **E)** Quantification of percent area stained red (indicating neutral lipids). **F)** Representative Masson’s Trichrome-stained liver sections; scale bar=200µm. Inset images (400x) correspond to boxed area in 100x images. **G)** Quantification of percent area stained blue (indicating collagen). Data represent means ± SEM, analyzed via 4-way ANOVA (factors: Cholesterol, Fat, Microbes, Time), followed by Tukey’s multiple comparisons. Bars with the same letter are not significantly different (*P*>0.05). n=4-7/group (see Table S4 for outliers). Low-fat (LF); Low-fat+high-cholesterol; Low-fat+very high-cholesterol (LFVHC); High-fat (HF); High-fat+high-cholesterol (HFHC); High-fat+very high-cholesterol (HFVHC).


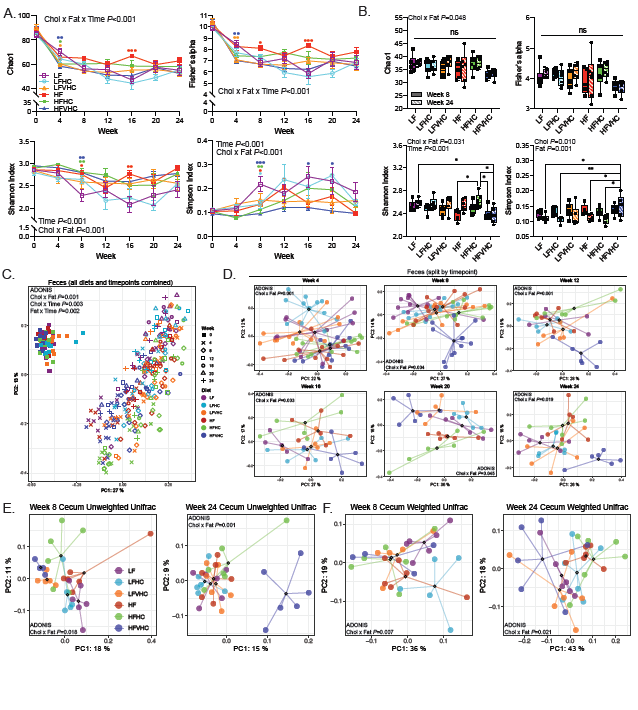


**Supplementary Figure 2. Dietary cholesterol and saturated fat differentially impact fecal and cecal microbiota diversity and composition over time in SPF mice.**

**A,B)** Chao1 (top left), Fisher’s alpha (top right), Shannon index (bottom left), and Simpson index (bottom right) α-diversity indices of fecal (A) and cecal (B) microbiota in SPF mice throughout the study (A). Data represent means ± SEM, analyzed via 3-way ANOVA (Factors: Cholesterol, Fat, Time) followed by Tukey’s multiple comparisons within timepoint. **P*<0.05, ***P*<0.01, ****P*<0.005. For fecal diversity indices, asterisk color represents significant differences from LF. **C,D)** Bray-Curtis β-diversity PCoA of fecal microbiota, analyzed via 3-way ADONIS (Factors: Time, Cholesterol, Fat) (C) or 2-way ADONIS (Factors: Cholesterol, Fat) (D). **E,F)** Unweighted (E) and Weighted (F) Unifrac β-diversity PCoA of cecal microbiota after 8 (left panels) and 24 (right panels) weeks on diet, analyzed via 2-factor ADONIS (Factors: Cholesterol, Fat). For all PCoA plots, dots represent individual mice, open circles represent centroids with lines connecting individual dots within a treatment group. n=12/group for fecal data weeks 0-8; n=6/group for fecal data weeks 12-24 (panels A,C,D). n=4-6/group (samples removed due to rarefaction; see Materials and Methods) for cecal data (panels B,E,F). Low-fat (LF); Low-fat+high-cholesterol (LFHC); Low-fat+very high-cholesterol (LFVHC); High-fat (HF); High-fat+high-cholesterol (HFHC); High-fat+very high-cholesterol (HFVHC).


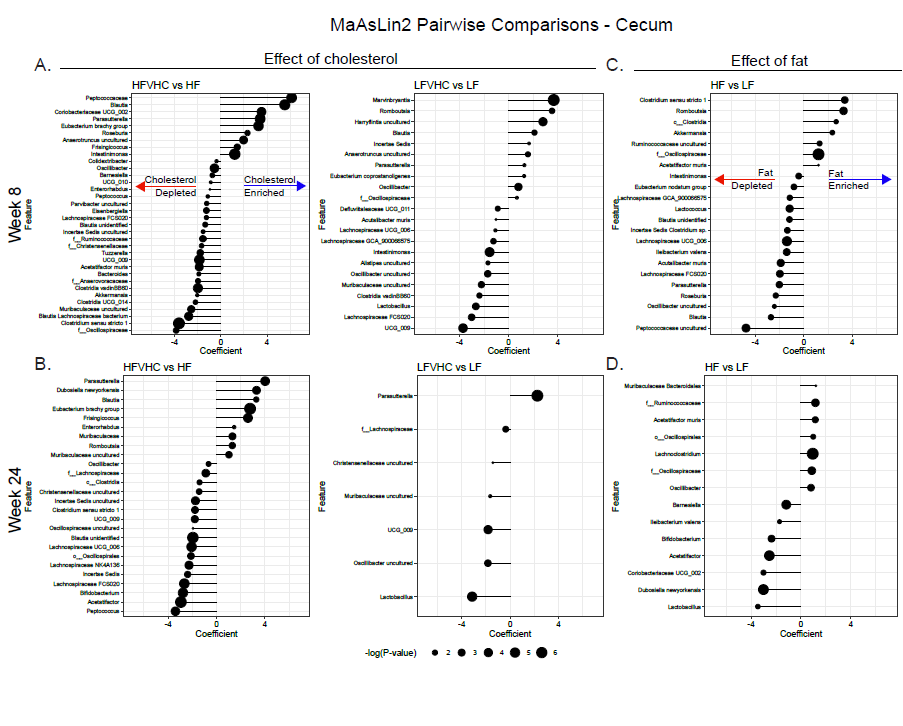


**Supplementary Figure 3. Distinct effects of dietary cholesterol and saturated fat on cecal microbiota composition at 8 and 24 weeks.**

**A-D)** Amplicon sequence variants (ASVs) significantly enriched or depleted by dietary cholesterol (A,B) or saturated fat (C,D) via MaAsLin2 pairwise comparisons at 8 (A,C) and 24 (B,D) weeks on diet. Positive coefficients indicate enrichment; negative coefficients indicate depletion. Dot size reflects -log(P-value). Taxonomic labels: “f__”: family-level annotation; “o__” order-level annotation; “c__” class-level. n=4-6/group. Low-fat (LF); Low-fat+very high-cholesterol (LFVHC); High-fat (HF); High-fat+very high-cholesterol (HFVHC).


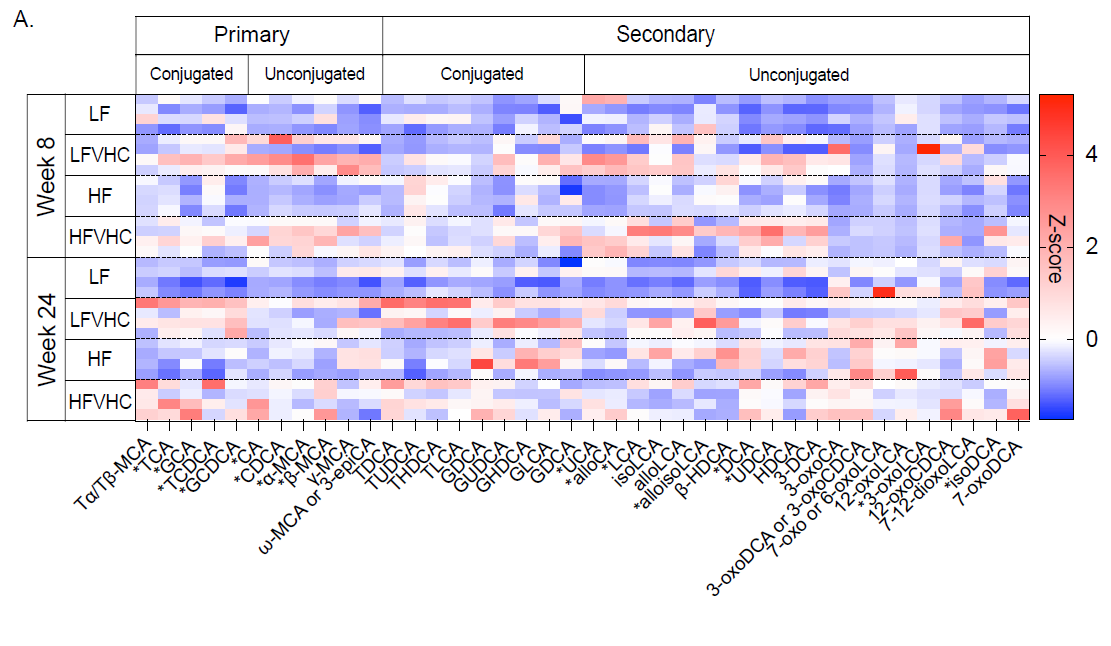


**Supplementary Figure 4. Fecal bile acid (BA) profiles are altered in response to dietary cholesterol and saturated fat.**

**A)** Heat map of fecal BA after 8 or 24 weeks in a subset (n=4/group) of Specific pathogen-free (SPF) mice, represented as Z-score of normalized peak area within each BA. BA with an asterisk(*) yielded quantitative values as shown in Figure 4G-L. Tauroα/β-muricholic acid (Tα/β-MCA); Taurocholic acid (TCA); Glycocholic acid (GCA); Taurochenodeoxycholic acid (TCDCA); Glycochenodeoxycholic acid (GCDCA); Cholic acid (CA); Chenodeoxycholic acid (CDCA); α/β/γ/ω-Muricholic acid (α/β/γ/ω-MCA); Taurodeoxycholic acid (TDCA); Tauroursodeoxycholic acid (TUDCA); Taurohyodeoxycholic acid (THDCA); Taurolithocholic acid (TLCA); Glycodeoxycholic acid (GDCA); Glycoursodeoxycholic acid (GUDCA); Glycolithocholic acid (GLCA); Glycodeoxycholic acid (GDCA); Ursocholic acid (UCA); Lithocholic acid (LCA); β-hyodeoxycholic acid (β-HDCA); Deoxycholic acid (DCA); Ursodeoxycholic acid (UDCA); Hyodeoxycholic acid (HDCA); n=4/group. Low-fat (LF); Low-fat+very high-cholesterol (LFVHC); High-fat (HF); High-fat+very high-cholesterol (HFVHC).


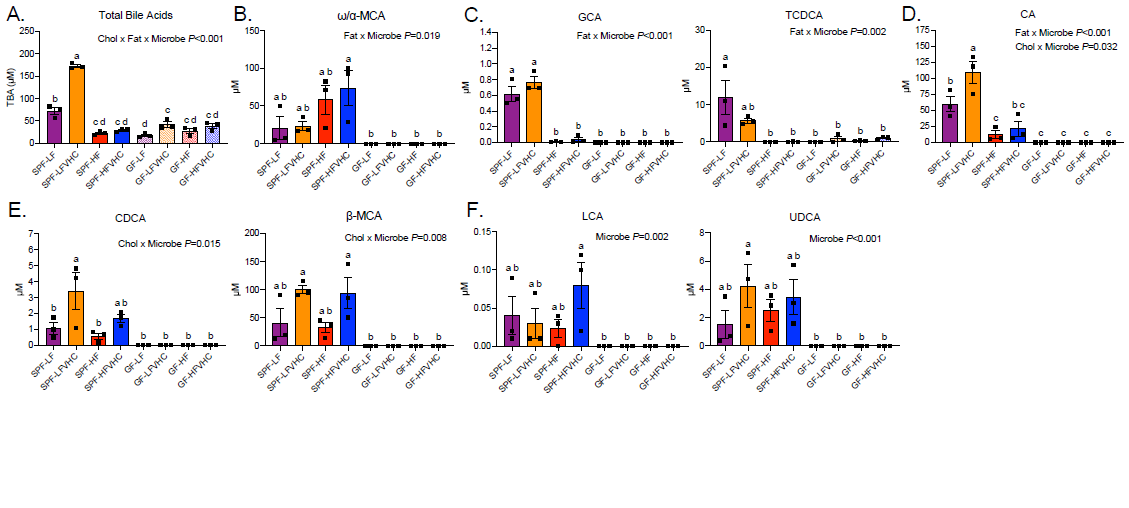


**Supplementary Figure 5: Total bile acid (TBA) concentration is not a primary mediator of HSC activation induced by cecal homogenates *in vitro*.**

**A)** Total bile acid (TBA) concentrations in cecal homogenates measured as in Figure 5A. **B-F)** Individual BA concentrations in cecal homogenates. Data represent means ± SEM, analyzed via 3-way ANOVA (factors: Cholesterol, Fat, Microbes) followed by Tukey’s multiple comparisons within timepoint. Bars with the same letter are not significantly different (*P*>0.05). n=3/group. α/β/ω-Muricholic acid (α/β/ω-MCA); Cholic acid (CA); Glycocholic acid (GCA); Taurochenodeoxycholic acid (TCDCA); Chenodeoxycholic acid (CDCA); Lithocholic acid (LCA); Ursodeoxycholic acid (UDCA); germ-free (GF); Specific pathogen-free (SPF). n=3/group.
